# New insights into the development and biological activity evaluation of mango products processed by sustainable emerging technologies

**DOI:** 10.7717/peerj.21235

**Published:** 2026-05-11

**Authors:** Adrián Domene-Vallero, Elena Rodríguez-Rodríguez, M. Luisa Ruiz del Castillo, Gracia P. Blanch, Begoña Olmedilla-Alonso, Lucía Giménez, Begoña de Ancos, Concepción Sánchez-Moreno

**Affiliations:** 1 Faculty of Pharmacy, Complutense University of Madrid (UCM), Madrid, Spain; 2 Institute of Food Science, Technology and Nutrition (ICTAN), Spanish National Research Council (CSIC), Madrid, Spain

**Keywords:** Mango (*Mangifera indica* L.), Mango by-products, Bioactive compounds, Functional beverages, Green extraction technologies, High-pressure processing (HPP)

## Abstract

Mango (*Mangifera indica* L.) processing generates large amounts of by-products, mainly peel and seed kernel, which are rich in phenolic compounds, carotenoids, vitamins, and dietary fiber. These bioactive compounds exhibit significant biological activities, including antioxidant, antidiabetic, anti-inflammatory, and anticancer properties, making them valuable for the development of functional foods. However, the efficient extraction of these bioactive compounds with minimal losses remains a challenge. This review explores the potential of incorporating mango by-products into the food industry, with a focus on their application in juice formulations to enhance nutritional and functional properties. It also discusses sustainable extraction techniques, including microwave-assisted extraction (MAE), ultrasound-assisted extraction (UAE), pressurized liquid extraction (PLE), supercritical fluid extraction (SFE), pulsed electric fields (PEF), high voltage electrical discharges (HVED), and enzyme-assisted extraction (EAE). Additionally, high-pressure processing (HPP) is discussed as a non-thermal alternative that preserves the integrity of bioactive compounds while ensuring microbial safety. The valorisation of mango by-products aligns with circular economy principles, offering a sustainable strategy to reduce waste and meet consumer demand for health-promoting, clean-label beverages.

## Introduction

As a member of the *Anacardiaceae* family with an origin traced to India, mango (*Mangifera indica* L.) has become a major fruit crop worldwide, being among the most cultivated and consumed fruits and ranking second in production only to bananas (FAO, 2024; Gupta et al., 2022). World production of mango and related fruits in 2023 was 61.11 million tons, with India leading the production with 26.24 million, followed by Indonesia (4.1 million tons) and China (3.9 million tons) (FAOSTAT, 2025). Pakistan, Mexico, and Brazil are also among the top producers, consolidating this tropical fruit as one of the most cultivated and in-demand globally. In 2024, global exports of mangoes and related fruits are anticipated to reach nearly 2.5 million tonnes, representing a 3% growth from the previous year, with major exporters are Mexico, Thailand, Peru and India. It is estimated that global imports of fresh mangoes will increase by 4% to 2.4 million tonnes in 2024, with the United States of America and the European Union maintaining as the two primary importers (FAO, 2024).

Mango is often referred to as “the king of fruits” valued for its characteristic aroma and its strong nutritional and phytochemical composition, with widely reported health-related properties (Lebaka et al., 2021; Espinosa-Espinosa et al., 2022). While commonly eaten fresh, it is also widely processed into products like juice, chutney, ice cream, powder, puree, and canned slices, with the production of pulp, juice, and nectar being its main industrial uses (Wall-Medrano et al., 2020; Oliver-Simancas et al., 2020). However, the industrial processing generates significant agro-industrial waste up to 60% of the fruit’s weight mainly in the form of peels, seed kernels and discarded fruits (Castro-Vargas et al., 2019; Wall-Medrano et al., 2020; Tacias-Pascacio et al., 2022).

In response to growing environmental concerns, food research is increasingly focused on the production of functional food and in the search of circular economy strategies that promote the reuse of these by-products. Studies have shown that mango by-products contain high levels of crucial nutrients and bioactive compounds such as phenolics, flavonoids, xanthones and carotenoids which have demonstrated antioxidant, anti-inflammatory, and anticancer properties (Aggarwal, Kaur & Bhise, 2017; Alañón et al., 2021a; Mirza et al., 2021; Gupta et al., 2022; Hu et al., 2023c; Mistry et al., 2023; Kaur, Panesar & Thakur, 2025). In particular, mango peel is a promising source of these compounds, with potential applications in food, pharmaceuticals, and nutraceutical industries (Sánchez-Camargo et al., 2019, 2021; Sánchez-Mesa et al., 2019; Alañón et al., 2021b). As the global population continuously aging, life expectancy rising and chronic diseases become more important, there is a growing demand for natural and healthy solutions to mitigate the risk of diseases associated with inflammatory processes, such as cancer, diabetes, neurodegenerative disorders and cardiovascular conditions (Gupta et al., 2022; Cuevas-Cianca et al., 2023; Mistry et al., 2023).

Among the diverse range of processed mango products, mango juice is the most popular processed product due to its appealing taste and nutritional value (Zhang et al., 2019). However, conventional thermal processing can degrade sensitive nutrients and volatile compounds, compromising quality. To address this, non-thermal technologies such as high-pressure processing (HPP), ultrasound-assisted extraction (UAE), and pulsed electric fields (PEF), among others, are being explored. These methods preserve nutritional and sensory attributes while reducing energy consumption. It was also projected that HPP-treated juice would play a leading role in the minimally processed food market, driven by increasing awareness of healthy eating, sustainability, and clean-label products (Bhattacharjee, Saxena & Dutta, 2019; Lou et al., 2022; Song et al., 2022).

The aim of this review is to evaluate how mango by-products can be leveraged in the food industry, with an emphasis on juice-based applications. Moreover, it highlights their nutritional and functional value, explores recent innovations in green extraction methods for bioactive compounds, and highlights sustainable processing technologies that contribute to both human health and environmental sustainability.

## Survey methodology

This review is designed for food scientists, process engineers, and researchers in food technology and nutraceuticals, particularly those focusing on sustainable processing and valorization of tropical fruit by-products.

This review provides a structured and comparative evaluation of advanced extraction and non-thermal technologies, including microwave-assisted extraction (MAE), ultrasound-assisted extraction (UAE), pressurized liquid extraction (PLE), supercritical fluid extraction (SFE), pulsed electric fields (PEF), enzyme-assisted extraction (EAE), and high-pressure processing (HPP), applied to different mango matrices (pulp, peel, and seed kernel). Previous reviews have explored the bioactive compounds in mango and the technologies used to extract them.

However, few studies provide a comprehensive, sustainable approach that integrates recovery from mango products and by-products with their potential application in functional beverages. In contrast to these earlier studies, this work emphasizes the valorization of mango by-products within a sustainability framework, integrating extraction efficiency, compound stability, and functional properties, with a particular focus on the development of functional beverages. By combining technological, compositional, and application-oriented perspectives, this review aims to provide a coherent roadmap for future research and industrial implementation. This integrated approach provides a more holistic and application-oriented framework than existing reviews, which typically focus either on mango composition, extraction technologies in general, or isolated aspects of by-product utilization.

A structured search strategy was implemented using Web of Science and Scopus, primarily covering 2020–2026 to capture recent developments, supplemented by seminal studies prior to 2020 for historical context. Search terms were organized into three conceptual blocks: (1) mango and by-products, (2) extraction/processing technologies, and (3) compositional and bioactivity outcomes (phenolics, carotenoids, antioxidant, anti-inflammatory, antidiabetic, anticancer properties, and functional beverage attributes). After screening titles and abstracts for relevance, approximately 250 publications were selected for detailed analysis.

Inclusion criteria targeted peer-reviewed studies applying sustainable or green technologies to mango matrices, reporting relevant compositional, technological, or biological outcomes. Exclusion criteria eliminated studies lacking methodological detail or unrelated to mango or bioactivity evaluation. Screening followed a staged process (title/abstract, then full-text), with ambiguous cases resolved through author consensus.

Data extraction captured standardized variables: matrix type, cultivar, processing conditions, target compounds, analytical methods, and key findings. Results were synthesized narratively and comparatively by technology class and outcome domain (extraction efficiency, stability, and biological activity), while acknowledging heterogeneity in study design and analytical approaches. Bias reduction strategies included multi-database searches, transparent eligibility criteria, duplicate removal, citation tracking, and balanced consideration of *in vitro*, *in vivo*, and human studies. Limitations related to variability in cultivars, matrices, and processing conditions were explicitly reported.

## Nutritional composition and bioactive components of mango (*Mangifera indica* L.)

**Figure 1:**
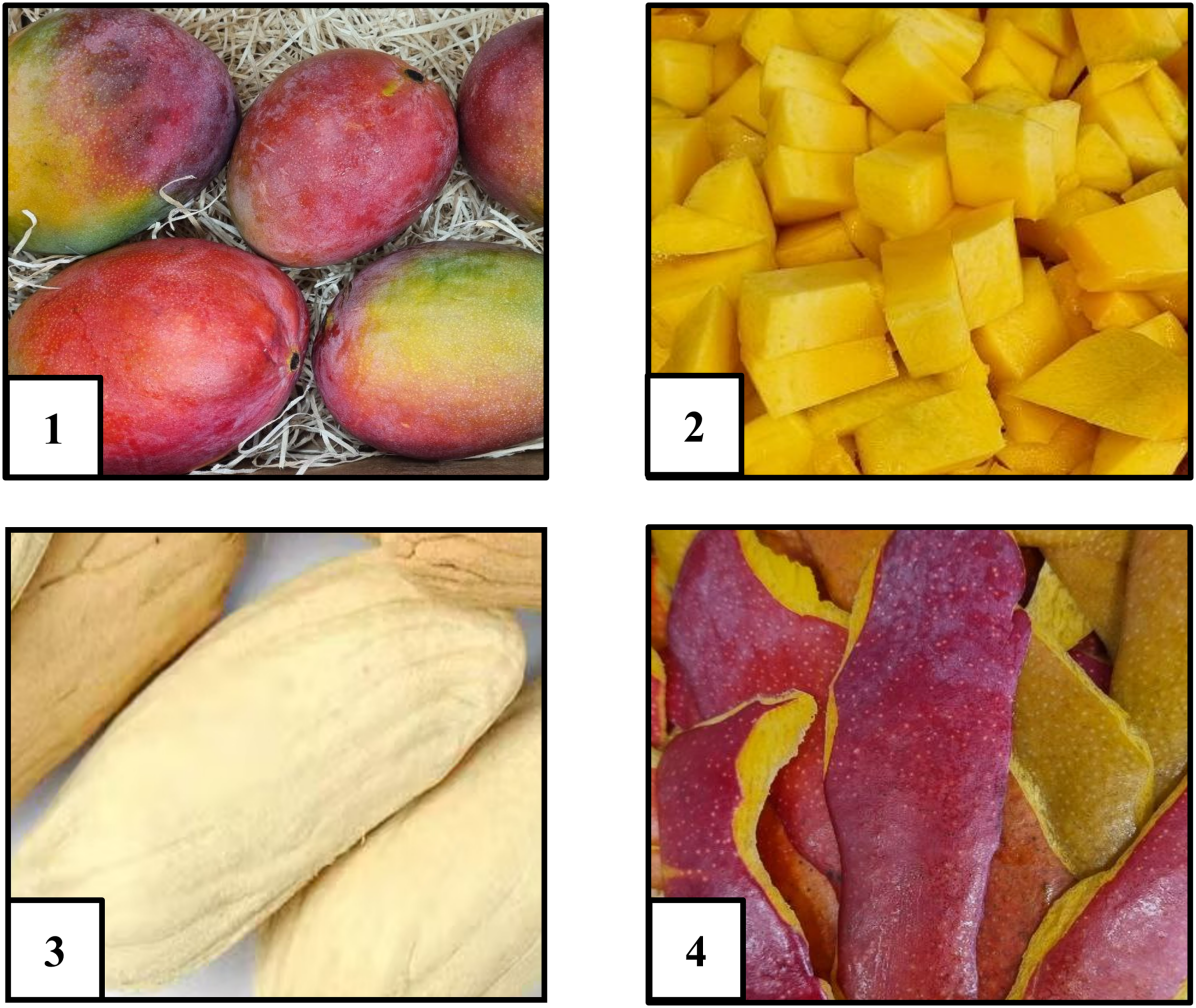
Anatomical components of mango (*Mangifera indica* L.): (1) whole fruit; (2) mesocarp (pulp); (3) endocarp (seed kernel); (4) exocarp (peel).

Mango fruit can be separated into pulp, peel, and seed kernel, corresponding to the mesocarp, epicarp, and endocarp, respectively (Fig. 1).

Among these anatomical parts, the pulp is the most widely consumed and industrially utilized component of the mango. It serves as the primary raw material for a variety of processed products such as juices, jams, purees, and fruit-based snacks, among others. The pulp is not only appreciated for its sensory attributes such as sweetness, aroma, and texture but also recognized as a rich source of essential nutrients and bioactive compounds, including carbohydrates, vitamins (notably A and C), carotenoid and phenolic compounds that contribute to its functional and nutritional value (Lebaka et al., 2021; Tacias-Pascacio et al., 2022; Takagi et al., 2025).

In contrast, mango peel represents approximately 15–25% of the fruit’s fresh weight, is typically discarded during the mango processing despite its high nutritional and functional potential (Rojas et al., 2020). Several studies have reported that mango peel contains significantly higher concentrations of polyphenolic and carotenoid compounds and other bioactive compounds compared to the pulp, making it a promising candidate for valorisation in functional food and nutraceutical applications (Serna-Cock, García-Gonzales & Torres-León, 2016; Lebaka et al., 2021; Kaur, Panesar & Thakur, 2024; Plaitho et al., 2024; Rodríguez-Rodríguez et al., 2025).

### Nutritional composition

Mango is considered a nutritionally valuable fruit due to its rich content of macro- and micronutrients, which vary depending on the cultivar and ripening stage (Torres-León et al., 2016; Lebaka et al., 2021). Based on USDA food composition data, Table 1 summarizes the nutritional composition of mango pulp, peel, and seed kernel for representative cultivars such as Tommy Atkins, Keitt, Kent, and Haden (U.S. Department of Agriculture (USDA), 2019).

**Table 1:** Nutritional profile of mango components.

Composition (Per 100 g)	Raw mango	Pulp	Peel	Seed kernel
Water (g)	83.5	78.9–82.8	72.5	9.1
Energy (kcal)	60	62.1–63.7	–	327
Carbohydrates, by difference (g)	14.98	16.20–17.28	28.2	18.2
Protein (g)	0.82	0.36–0.40	3.6	6.61
Total lipid (g)	0.38	0.30–0.53	2.2	9.4
Ash (g)	0.36	0.34–0.52	0.6–3.0	–
Total dietary fiber (g)	1.6	0.85–1.06	40–72.5	70
Sugar (g)	13.7	10.7–11.1	25	2.8
Minerals (mg)				
Calcium (Ca)	11	7–16	60–87.5	450
Iron (Fe)	0.16	0.09–0.41	40.6	11.9
Magnesium (Mg)	10	8–19	100	100
Phosphorus (P)	14	10–18	–	140
Potassium (K)	168	120–211	444	365
Sodium (Na)	1	0–3	107	150
Zinc (Zn)	0.09	0.06–0.15	1.74	1.1
Copper (Cu)	0.111	0.04–0.32	10.4	–
Manganese (Mn)	0.063	–	0.25	–
Selenium (Se)	0.6	0-0.6	–	–
Vitamins				
Vitamin C (total ascorbic acid, mg)	36.4	13.2–92.8	18–257	17
Thiamin (mg)	0.028	0.01–0.04	–	0.08
Riboflavin (mg)	0.038	0.02–0.07	–	0.13
Niacin (mg)	0.669	0.2–1.31	–	0.19
Pantothenic acid (mg)	0.197	0.16–0.24	–	0.12
Vitamin B_6_ (mg)	0.119	0.05–0.16	–	–
Folate, dietary folate equivalents (µg)	43	20–69	–	–
Vitamin A, retinol activity equivalents (µg)	54	54	100	–
Vitamin E (α-tacopherol) (mg)	0.9	0.79-1.02	0.25–0.59	1.3
Vitamin K (phylloquinone) (µg)	4.2	4.2	–	59

**Note:**

Sources: U.S. Department of Agriculture (USDA) (2019), Zafar & Sidhu (2017), Lebaka et al. (2021), Gupta et al. (2022).

Mango is acknowledged as a significant source of macro- and micronutrients. Its pulp predominantly comprises 16–18% carbohydrates, together with proteins, amino acids, lipids, organic acids, and dietary fiber. Additionally, it supplies essential minerals such as calcium (Ca), magnesium (Mg), and iron (Fe), along with vitamins A, C, E, and K (Maldonado-Celis et al., 2019). As shown in Table 1, the mango peel offers even higher concentrations of carbohydrates (20–30%), proteins, amino acids, lipids, organic acids, and dietary fibre, highlighting its potential as a functional ingredient. Similarly, the mango seed kernel (MSK) demonstrates superior nutritional density, with elevated levels of carbohydrates, lipids, protein and various minerals compared to the pulp (Torres-León et al., 2016).

### Macronutrients

Mango offers a wide range of macronutrients, the composition of which varies depending on the cultivar and ripening stage (Dar et al., 2016; Maldonado-Celis et al., 2019). Mango ripening is generally accompanied by the conversion of reserve carbohydrates into soluble sugars, resulting in higher levels of fructose, glucose, and sucrose in ripe fruit, while unripe mango remains comparatively enriched in starch and pectin (Gupta et al., 2022). Similar ripening-associated rises in mono- and disaccharides have been described for many cultivars (Drouillard et al., 2023; Liu et al., 2023a).

Mango pulp contains significant quantities of pectin, an important structural carbohydrate and a critical component in gelling sugar. While pectin accumulates in immature fruit, its molecular weight decreases as ripening progresses, primarily due to hydrolytic activity of pectin-degrading enzymes (Gupta et al., 2022). Mango pulp contains 16–18% of carbohydrates (Lemmens et al., 2013) while mango peel contains between 20–30% (Ajila & Prasada Rao, 2013). Mango seeds have a higher carbohydrate content than pulp but a lower content than peel (Table 1), producing 21% pure starch (Torres-León et al., 2016). Studies have shown that starch undergoes hydrolysis into simple sugars, primarily fructose and glucose, during mango ripening (Kaur, Panesar & Thakur, 2024).

Mango, like most fruits, contains relatively little protein compared to its other macronutrients. Moreover, the protein level in mangoes depends on the cultivar and the region of cultivation (Maldonado-Celis et al., 2019). For instance, mangoes from Peru display a total protein content ranging from 1.5% to 5.5%, while in Colombia, the mango pulp contains only 0% to 0.6% protein (Dar et al., 2016; Maldonado-Celis et al., 2019). Analysing the protein levels in different parts of the mango (Table 1) reveals that the seed kernel is the richest in protein, whereas the pulp has the lowest content. Notably, the mango peel exhibits a higher protein content than the pulp, making it a valuable source of both protein and essential amino acids.

The amino acid composition also varies among cultivars and maturation levels (Maldonado-Celis et al., 2019). In the ripe state, amino acids such as alanine, arginine, glycine, serine, leucine, and isoleucine are present in considerable amounts, while all other amino acids occur only in trace amounts. Table 2 shows the content of amino acids in raw peeled mango (U.S. Department of Agriculture (USDA), 2019). A compositional analysis of mango seeds expressed per 100 g of protein identified several essential amino acids. Reported values were 1.2 g methionine, 2.7 g tyrosine, 3.4 g phenylalanine, 3.4 g threonine, 4.3 g lysine, 4.4 g isoleucine, 5.8 g valine, and 6.9 g leucine (Abdalla et al., 2007).

**Table 2:** Aminoacid composition in edible portion of mango fruit (U.S. Department of Agriculture (USDA), 2019).

Composition (Per 100 g)	Pulp
Aminoacids (mg)	
Tryptophan	13
Threonine	31
Isoleucine	29
Leucine	50
Lysine	66
Methionine	8
Phenylalanine	27
Tyrosine	16
Valine	42
Arginine	31
Histidine	19
Alanine	82
Aspartic acid	68
Glutamic acid	96
Glycine	34
Proline	29
Serine	35

Lipids and fatty acids are often highlighted for their caloric contribution and their technological functionality in food matrices (Nadeem, Imran & Khalique, 2016; Khan et al., 2018; Gupta et al., 2022). However, in mango, these compounds are not primarily concentrated in the pulp. As shown in Table 1, the pulp contains comparatively little fat: its lipid fraction is smaller than the protein fraction and dwarfed by the carbohydrate content. A clear gradient is also evident across fruit tissues, with higher lipid levels in the peel and the greatest accumulation in the seed fraction (Table 1). Consistent with this distribution, mango seeds are a notable reservoir of oil, typically ranging from 8.15% to 13.16%, and have been proposed as an attractive source of nutritionally relevant lipids with potential health-promoting properties (Solís-Fuentes & Durán-de-Bazúa, 2011; Gupta et al., 2022).

In general, organic acids show mild acidity, while their molecular weights differ markedly depending on the specific structure. These acids play a vital role in imparting fruits with their characteristic tastes and flavors, thereby influencing their overall organoleptic quality (Vallarino & Osorio, 2019; Lebaka et al., 2021; Gupta et al., 2022). In mango pulp, several organic acids have been identified, including citric, malic, oxalic, succinic, ascorbic, and tartaric acids (Lebaka et al., 2021).

Mango pulp serves as an energy-dense food, providing between 60 and 190 kcal per 100 g of fresh fruit (Gupta et al., 2022). Its high-water content ranging from 75% to 85% works in tandem with an array of essential nutrients (Zafar & Sidhu, 2017; Maldonado-Celis et al., 2019; U.S. Department of Agriculture (USDA), 2019; Lebaka et al., 2021; Gupta et al., 2022). In addition to these features, both soluble and insoluble dietary fibers contribute significantly to its nutritional profile. Among various cultivars, soluble fibers vary between 16% and 28%, whereas insoluble fibers range from 29% to 50% (Ajila & Prasada Rao, 2013; Zafar & Sidhu, 2017; Maldonado-Celis et al., 2019; U.S. Department of Agriculture (USDA), 2019; Lebaka et al., 2021; Gupta et al., 2022; Silva et al., 2022). It is noteworthy that raw mangoes tend to have lower levels of soluble fiber compared to when they are fully ripe. Moreover, the peel emerges as a particularly fiber-rich component, containing between 28% and 78% total dietary fiber with insoluble fractions accounting for 14% to 50% and soluble fractions for 13% to 28% of the total (Serna-Cock, García-Gonzales & Torres-León, 2016; Zafar & Sidhu, 2017; Maldonado-Celis et al., 2019; U.S. Department of Agriculture (USDA), 2019; Lebaka et al., 2021; Gupta et al., 2022).

### Micronutrients: vitamins and minerals

Vitamins and minerals are considered to be critical micronutrients that are present in mango pulp and its by-products (Pant et al., 2020; Lebaka et al., 2021). As shown in Table 1, the vitamin profile of mango pulp includes both fat- and water-soluble compounds. Notably, USDA data report higher levels of vitamin C and provitamin A-related vitamin A activity (linked to β-carotene and β-cryptoxanthin) compared with vitamins B, E, and K (U.S. Department of Agriculture (USDA), 2019). Vitamin C concentrations show substantial variability, ranging from 98 mg up to 18 g/kg, which supports the idea that mango can contribute significantly to fulfilling vitamin-related dietary needs when consumed regularly (World Health Organization, 2003). Vitamin C functions as an antioxidant and immune booster, playing a crucial role in collagen regeneration, the prevention of scurvy, and iron absorption (Diplock et al., 1998; Khadim & Al-Fartusie, 2021; Gupta et al., 2022). During mango ripening, ascorbic acid (vitamin C) content tends to decrease, so less mature fruits usually show higher concentrations than fully ripe ones (Matheyambath, Subramanian & Paliyath, 2016). This reduction has been linked to ripening-associated biochemical routes, including ethylene-related metabolism and the formation of oxalate and tartrate (Singh et al., 2011). A plausible explanation is that vitamin C is actively utilized as a coenzyme in multiple enzymatic reactions, which can contribute to its progressive depletion as metabolism intensifies. The USDA has reported an average of 36.4 milligrams per 100 g of vitamin C in mango pulp (Table 1) (U.S. Department of Agriculture (USDA), 2019). Beyond its role in vision, vitamin A together with its metabolites has been related to immune regulation and antioxidant effects and has also been discussed in the context of reduced risk for cancer and cardiovascular conditions. Consuming one fresh mango (about 250–300 g) provides an estimated 10–12% of the retinol RDA, given that vitamin A in the pulp has been reported in the 1,000–6,000 IU range (Lebaka et al., 2021). The ingestion of mango is considered to be an effective method of preventing vitamin A deficiency (Muoki et al., 2009; Matheyambath, Subramanian & Paliyath, 2016; Lebaka et al., 2021). Relative to vitamin A, mango pulp generally contains smaller amounts of vitamins E and K, and both tend to increase during ripening. However, some Indian cultivars (*e.g*., Deshahari) have been reported to retain comparatively low vitamin E levels even at maturity (Singh et al., 2011). In fresh pulp, vitamin E is mainly present as α-tocopherol, at about 1.3 mg per 100 g (Robles-Sánchez et al., 2009). Variations in vitamin E may partly reflect the role of vitamin C in generating the tocopheroxyl radical, a key intermediate in tocopherol redox cycling and the regeneration of the active form (Mène-Saffrané, 2018). Accordingly, vitamin C and vitamin E often show an inverse relationship during fruit development. Moreover, ripe mango peel has been found to contain more vitamin E than unripe peel (Gupta et al., 2022). In mango, the B-vitamin group comprises water-soluble enzyme cofactors and related derivatives that participate in multiple metabolic processes in both plants and humans. Depending on maturity, mango pulp contains approximately 1.5–2.5 mg/100 g of B-complex vitamins in fresh tissue (Dar et al., 2016). With the exception of biotin (vitamin B7), the remaining B vitamins are generally present in the pulp (Gupta et al., 2022).

Mango pulp contributes a broad spectrum of essential minerals involved in numerous biochemical processes. As summarized in Table 1, its mineral profile includes macrominerals and trace elements such as Ca, Na, Cu, K, Fe, P, Mn, Mg, Zn, as well as B (0.6–10.6 mg/kg) and selenium (Lebaka et al., 2021). Nevertheless, mineral concentrations are generally higher in the peel than in the pulp, with potassium (K) being the main exception to this pattern (Table 1).

### Phenolic compounds

Plants orchestrate the synthesis of a diverse class of secondary metabolites known as phenolic compounds through finely tuned metabolic pathways. These routes utilize precursors as phosphoenolpyruvate from glycolysis and erythrose-4-phosphate from the pentose phosphate pathway demonstrating a sophisticated interplay between primary metabolism and specialized biosynthetic mechanisms (Tacias-Pascacio et al., 2022). As a result, researchers have catalogued over 8,000 distinct structural types, and more than 50,000 individual molecules have been identified within this group (Alara, Abdurahman & Ukaegbu, 2021; Ávila-Román et al., 2021). This remarkable diversity is an integral part of the human diet due to their significant biological functions and associated health benefits (Burton-Freeman, Sandhu & Edirisinghe, 2017; Gupta et al., 2022).

Phenolic compounds can be grouped into various categories such as phenolic acids, phenolic alcohols, flavonoids, lignans, stilbenes, and tannins (Das et al., 2020; Tacias-Pascacio et al., 2022). Nevertheless, the most widely accepted classification divides them into two primary classes: flavonoid and non-flavonoid compounds (Singh et al., 2016). This binary division not only simplifies the vast complexity inherent in their structures but also serves as a conceptual framework for understanding their varied bioactivities.

Flavonoids are compounds which structure is characterized for having two aromatic rings linked by a three-carbon bridge (C6-C3-C6). This core structure diversifies into subgroups such as flavones, flavonols, flavan-3-ols, flavanones, flavanonols, isoflavones, chacones and anthocyanins (Zhou et al., 2020). In their natural state, flavonoids are commonly encountered as glucosides, this modification can significantly influence in their properties and overall bioavailability (González-Paramás et al., 2019). In contrast, non-flavonoid phenolic compounds predominantly consist of metabolites classified as phenolic acids, which are further divided into hydroxybenzoic and hydroxycinnamic acids (Jesus et al., 2022).

Phenolic compounds have gained significant attention due to their wide variety of biological activities, including antioxidant, anti-inflammatory, antimutagenic, anticancer, antitumor, antimicrobial, and even cytotoxic effects (Brito, Ferreira & Fai, 2022). Their bioactivity is largely attributed to the unique characteristics of their chemical structure enable precise interactions with cellular proteins, enzymes, and receptors, thereby modulating critical biological processes (Vuolo, Lima & Maróstica Junior, 2019). As such, these compounds serve as key actors in both preventive nutrition and the potential development of novel therapeutic agents.

**Figure 2:**
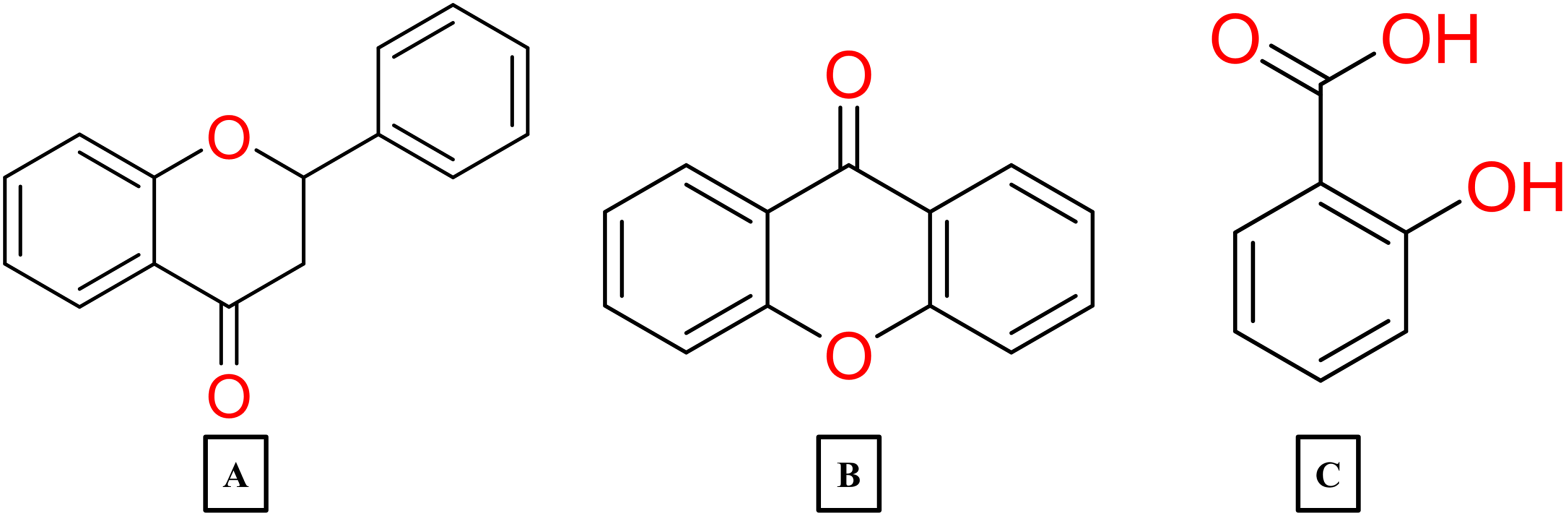
Basic skeleton structure of: (A) flavonoids; (B) phenolics acids; (C) xanthones.

**Figure 3:**
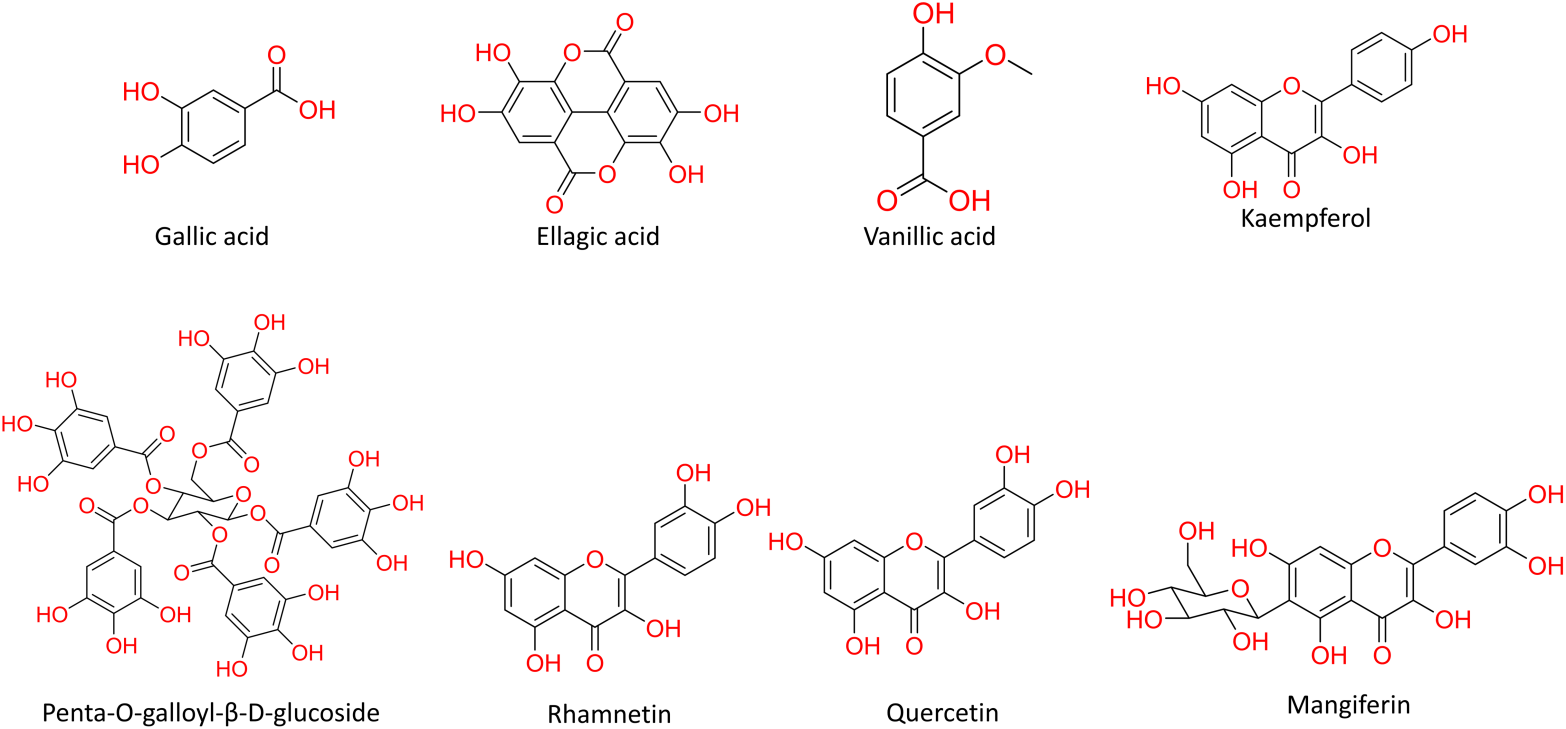
Major phenolic compounds in mango: structural overview (Mirza et al., 2021).

Turning to the mango fruit, multiple studies have reported that every component of the mango fruit contains considerable amounts of phenolic compounds, such as flavonoids, phenolic acids, benzophenones, xanthones (mangiferin) and gallatos and gallotannins (Gutiérrez-Sarmiento et al., 2020; Tacias-Pascacio et al., 2022) (Fig. 2). In particular, compounds such as gallic acid, ellagic acid, mangiferin, quercetin derivatives and gallotannins as penta-O-galloyl-ß-glucoside are among the most abundant phenolic compounds in various mango by-products, where the peel contains the highest concentrations of phenolic compounds (Lebaka et al., 2021; Tacias-Pascacio et al., 2022) (Fig. 3). However, it is important to note the importance of variables that can vary considerably the concentration and composition of phenolic compounds, like the mango cultivar, the cultivation region, postharvest handling, maturity stage and the specific part of the fruit that is analysed (Maldonado-Celis et al., 2019).

Mango pulp, celebrated for its sensory appeal and bioactive profile, is predominantly processed into juices, nectars, and jellies. However, even gentle processing can subject the pulp to physicochemical changes triggered by endogenous enzymatic activity, potentially losing its organoleptic qualities (Varoquaux & Wiley, 2017). By contrast, mango peel has gained considerable interest of researchers, due to its biological composition, particularly the high content of different bioactive compounds such as polyphenols, carotenoids, tocopherols and ascorbic acid (Chen et al., 2019). Particularly, the high content of phenolic compounds gives it a great interest for the pharmaceutical and food industries. Studies indicate that mango peel not only contain higher concentrations of phenolic compounds than other fruit peels but also surpass the phenolic content found in mango pulp (Lebaka et al., 2021; Tacias-Pascacio et al., 2022).

The effectiveness of mango peel as a source of phenolics is intricately linked to the processing methods employed. Pretreatment and preservation techniques play a critical role in either liberating intracellular bioactives or inadvertently triggering their degradation (Tacias-Pascacio et al., 2022). Moreover, as in the pulp, in mango peel it has also been determined that during the ripening of the fruit, the phenolic compounds content increases to a certain point and subsequently declines (Vithana, Singh & Johnson, 2018). In one study its documented that the phenolic compounds content present in peels of three mango cultivar (Badami, Raspuri and Kensington Pride) decreased during fruit ripening (Supriya, Kumar & Kudachikar, 2020). However, an extrinsic factor with a great effect on the extraction yield of phenolic compounds is the method used for this extraction (Rebollo-Hernanz et al., 2021).

### Chlorophylls and carotenoids

As mango develops, the relative abundance of major pigments changes, leading to the characteristic colour shift observed during ripening. The fruit is usually green when immature and becomes yellow to orange at full ripeness, with noticeable cultivar-to-cultivar variation that can be used to infer quality. In parallel, texture also evolves in both peel and pulp. These ripening patterns are linked to chlorophyll degradation and the accumulation of carotenoids and flavonoids, reflecting underlying metabolic adjustments (Sudhakar, Latha & Reddy, 2016).

Mango colour development during ripening is largely explained by a changing pigment balance. Immature fruit appears green due to chlorophylls chlorophyll a (blue-green) and chlorophyll b (yellow-green), typically at ~3:1 (Hui & Sherkat, 2005; Maldonado-Celis et al., 2019; Lebaka et al., 2021). As ripening proceeds, chlorophyll declines in parallel with thylakoid disassembly in chloroplasts, a process promoted by ethylene *via* induction of peel chlorophyllase biosynthesis (Choo, 2019). Concurrently, carotenoids (notably β-carotene) accumulate in both pulp and peel, driving the cultivar-dependent shift toward yellow, orange, or red hues (Ketsa, Phakawatmongkol & Subhadrabhandhu, 1999; Lebaka et al., 2021; Choo, 2019; Maldonado-Celis et al., 2019).

**Figure 4:**
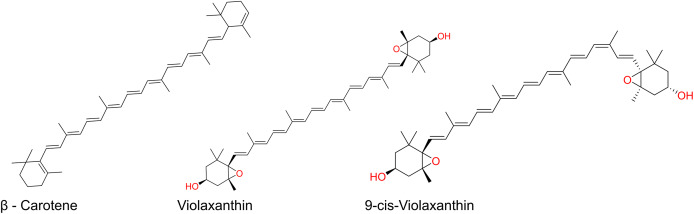
Major carotenoids in mango.

Carotenoids are among the main pigment families found in mango, and mango is often described as a particularly carotenoid-rich fruit. These compounds constitute a broad class of naturally occurring organic pigments distributed across plants and many other organisms (Kultys & Kurek, 2022). In mango, carotenoids largely shape the colour of both peel and flesh, giving rise to yellow, orange, and red tones; they are stored in chromoplasts, although their visual contribution may be partially obscured by chlorophyll in certain tissues (Choo, 2019). The carotenoid pool in mango can be grouped into (i) carotenes, non-oxygenated hydrocarbons such as α-, β-, and γ-carotene, and (ii) xanthophylls, which are oxygenated derivatives including auroxanthin, antheraxanthin, neoxanthin, lutein, violaxanthin, cryptoxanthin, and zeaxanthin. Overall, about 25 distinct carotenoids have been reported in mango peel and pulp (Lebaka et al., 2021). Within this profile, all-trans-β-carotene is typically the dominant species (≈60% of total carotenoids), followed by violaxanthin isomers (all-trans and 9-cis), as summarized in Fig. 4 (Varakumar, Kumar & Reddy, 2011; Ediriweera, Tennekoon & Samarakoon, 2017; Maldonado-Celis et al., 2019; Lebaka et al., 2021).

Mango pulp contains carotenoids with significant variability among varieties. For example, β-carotene ranges from 580 to 3,558 µg/100 g of edible portion. β-carotene is present in all varieties reviewed, while β-cryptoxanthin has been reported in Tommy Atkins and Keitt, and lycopene quantified in Tommy Atkins. Other carotenoids such as neoxanthin and violaxanthin are also described in certain varieties (Dias et al., 2018). The carotenoid content is directly affected depending on the fruit maturity stage and the environmental conditions during the fruit growth (Bramley, 2013). Ellong, Adenet & Rochefort (2015) evaluated the variation in total carotenoid levels between unripe and ripe fruits across four mango cultivars. They reported that total carotenoids of mango cultivars Bassignac, Green, Julie, and Moussache were between 276.17 μg (Green) and 2,183 μg (Bassignac) per 100 g fresh weight in the unripe stage. These values increased at the ripe stage, between 603.35 μg (Moussache) and 4,138.50 μg (Bassignac) of total carotenoids per 100 g fresh weight (Ellong, Adenet & Rochefort, 2015). A similar profile was observed in other study where they studied the carotenoid content of 12 mango cultivars from Bangladesh at three stages (Haque et al., 2015). These studies indicate that the carotenoid content increased from green into ripe stage. Additionally, carotenoids are found in higher concentrations in the peel than in the pulp (Rodríguez-Rodríguez et al., 2025).

### Volatile aroma compounds

In mango, volatile aroma compounds (VACs) are low-molecular-weight constituents (<400 Da) with diverse functional groups, present either as free volatiles or as glycosidically bound forms (Maldonado-Celis et al., 2019; Gupta et al., 2022). Their high vapour pressure supports rapid dispersion in air, water and soil, and they are notably biodegradable (Sharifi & Ryu, 2018; Oliver-Simancas et al., 2020; Gupta et al., 2022; Silva et al., 2022). Although usually found only at trace levels (~50 ppm or below), VACs largely define mango aroma and include terpenes (mono- and sesquiterpenes), esters, lactones, alcohols, aldehydes, ketones, volatile fatty acids, carotenoid-derived volatiles and phenolic degradation products (Pino et al., 2005; Li et al., 2017; Cuevas-Glory et al., 2020).

There is a heterogeneous quantitative and qualitative distribution of VACs among different cultivars, maturity stages, and fruit tissue (Maldonado-Celis et al., 2019; Thiruchelvam, Landahl & Terry, 2020). In mango fruit, nearly contains 400 distinct VACs have been identified. Among these, 3-carene is the most predominant in most cultivars and is mainly responsible for the characteristic mango aroma, while limonene, β-ocimene, myrcene and α-terpinolene are prominent in other cultivars (Pino et al., 2005; Lebrun et al., 2008; Thiruchelvam, Landahl & Terry, 2020; Gupta et al., 2022). Thiruchelvam, Landahl & Terry (2020) reported that while the peel and pulp share similar volatile composition, the concentrations are generally higher in the peel. This difference is likely due to a greater presence of unbound volatile compounds, such as aldehydes, fatty acids, and terpenes in the peel compared to the pulp. Additionally, Lalel et al. (2003) reported that in the Kensington Pride cultivar, glycosidically-bound volatile compounds are more abundant in the peel than in the pulp during the mature and half-ripe stages relative to the fully ripe stage.

## Extraction of phenolic and carotenoid compounds of mango products

Efficient extraction of phenolics, carotenoids, and related bioactives underpins their identification and quantification in fruit and vegetable matrices. An optimal method should be quantitative, non-destructive, and time-efficient (Chávez-González et al., 2020; Gupta et al., 2022). Given the wide chemical diversity of these compounds, standardized and integrated screening strategies are needed to target fractions with potential health relevance. Robust characterization therefore requires an extraction approach that recovers multiple compound classes while limiting co-extracted interferents (Ballesteros-Vivas et al., 2019; Chávez-González et al., 2020; Martínez-Ramos et al., 2020; Sánchez-Camargo et al., 2021; Gupta et al., 2022).

Traditionally, the extraction of phenolic and carotenoid compounds was performed using conventional or traditional methods, such as soxhlet extraction, decotion, serial exhaustive extraction, digestion, maceration, percolation and infusion (Alara, Abdurahman & Ukaegbu, 2021; Tacias-Pascacio et al., 2022). Many laboratories favour these conventional techniques because of their ease to use and low cost. However, studies have shown that these methods present environmental problems and exhibit low efficiency of these conventional due to the requirement for large amounts of organic solvents due to the rigid structure of cell walls of microorganisms. Conventional extraction approaches typically culminate in an evaporation-based concentration step after recovery, adding substantial time to the process and often involving solvents that are unsuitable for food applications, including hexane, acetone, and methanol (Alara, Abdurahman & Ukaegbu, 2021; Marçal & Pintado, 2021).

**Figure 5:**
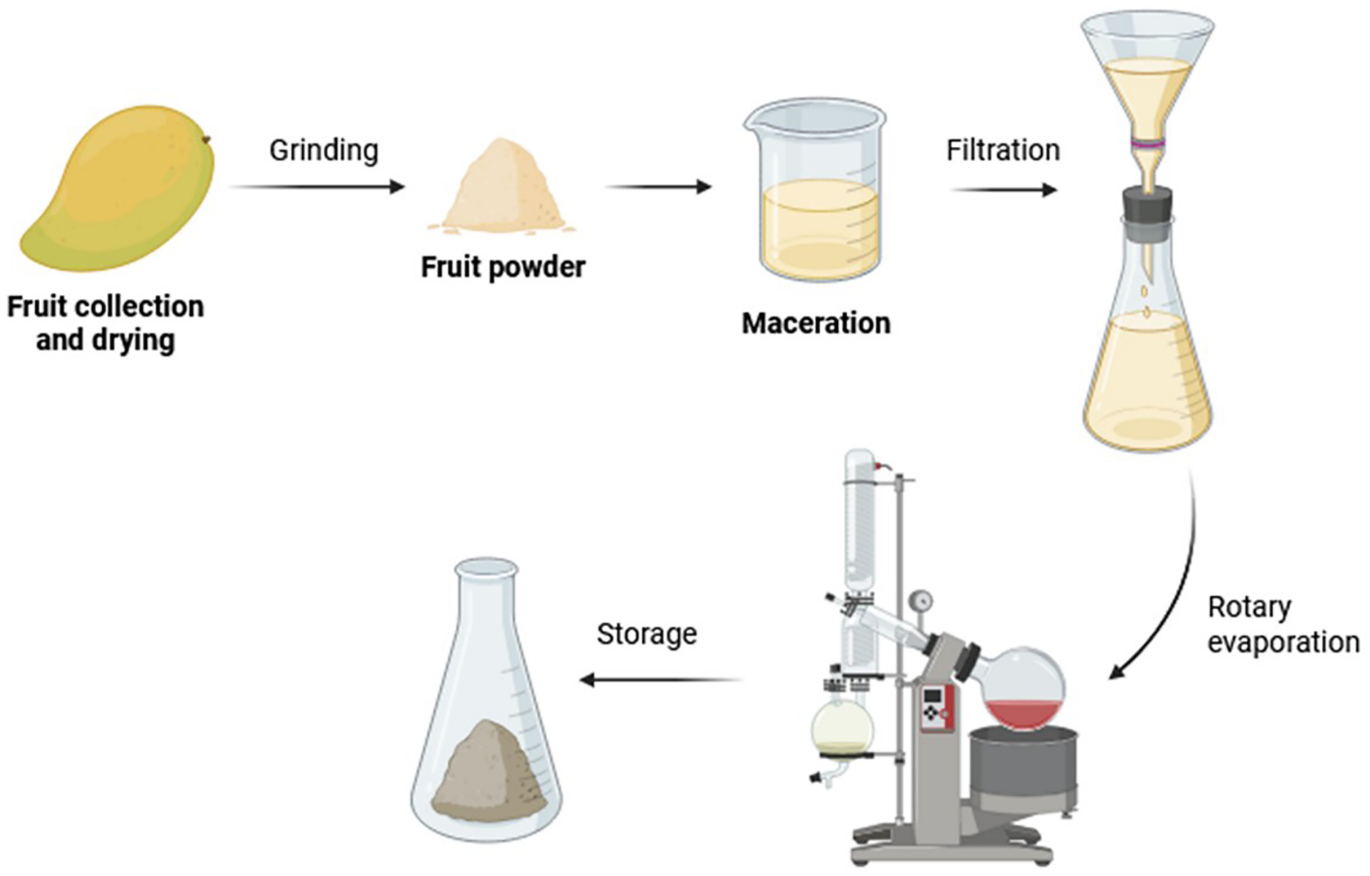
General scheme of the maceration-based extraction process from fruit by-products.

Among these traditional methods, solid-liquid extraction, commonly known as maceration is the most frequently used technique for recovering phenolic compounds from vegetable (Tacias-Pascacio et al., 2022). This process is relatively simple and involves immersing a dried sample in a solvent such as ethanol, methanol, acetone, or ethyl acetate at varying concentrations. There are also different types of maceration techniques, including cryogenic maceration using solid CO_2_, and enzymatic maceration with pectinase or other enzymes under constant stirring and controlled temperature conditions (Olejar, Fedrizzi & Kilmartin, 2015; Safdar, Kausar & Nadeem, 2017; Coelho et al., 2019). After maceration, the liquid phase containing the concentrated extracts is separated from the solid residue, as illustrated in Fig. 5 (Ćujić et al., 2016). For carotenoid extraction, it is common to perform an additional maceration of the residual solid in order to maximize the overall extraction yield using a fresh portion of solvent. This step typically employs organic solvents such as hexane, acetone, dichloromethane, chloroform, or ethanol, due to the moderate to high hydrophobicity of carotenoids (Yara-Varón et al., 2016). Nonetheless, maceration share the main drawbacks of the conventional methods as they are mention before of requiring large solvent volumes and extended extraction times (Alara, Abdurahman & Ukaegbu, 2021).

The growing interest in phenolic compounds is largely attributed to their biological activities, notably their antioxidant capacity. Extraction parameters such as temperature, solvent, mass:solvent ratio (g:mL), among others, can significantly influence not only their yield but also their bioactivity of the extracted compounds. For this reason, it is important to study the effect that the extraction conditions have not only on the quantity of extracted phenolic compounds, but also in their bioactivity. Therefore, optimizing these parameters is crucial to maximize the beneficial properties of the extracts.

In response to the limitations of conventional methods, recent studies have started the development of several green methods aimed to reduce the extraction time and energy consumption, as well as the possibility of using safer and more sustainable solvents (Tacias-Pascacio et al., 2021). Green extraction technologies such as ultrasound-assisted extraction (UAE) and microwave-assisted extraction (MAE) have been successfully applied to recover bioactive compounds from mango peels in an eco-friendly manner (Sánchez-Camargo et al., 2021; Villacís-Chiriboga et al., 2021; Tacias-Pascacio et al., 2022). These methods minimize solvent use, reduce energy consumption, and preserve the integrity of heat-sensitive compounds, making them particularly suitable for industrial-scale applications in juice production (Alara, Abdurahman & Ukaegbu, 2021; Gil-Martín et al., 2022).

The valorization of mango peel and other agro-industrial by-products through these green technologies aligns with the principles of the circular economy, transforming agro-industrial waste into value-added ingredients (Serna-Cock, García-Gonzales & Torres-León, 2016; Castro-Vargas et al., 2019). This approach not only reduces the environmental burden associated with organic waste disposal but also contributes more sustainable food systems (Gupta et al., 2022).

In addition to UAE, MAE and PLE, other innovative techniques such as supercritical fluid extraction (SFE), pulsed electric fields (PEF) and high voltage electrical discharges (HVED), enzyme assisted extraction (EAE), or even a combination of these techniques, have shown promising results. Overall, emerging green extraction approaches tend to outperform conventional methods by reducing solvent demand and processing time while improving extraction efficiency and reproducibility, and by limiting the generation of harmful residues. Beyond these technical benefits, their adoption supports the valorization of mango waste and other plant by-products as sources of extracts for food, pharmaceutical, and nutraceutical applications (Tacias-Pascacio et al., 2022). The next sections provide an overview of these alternative extraction strategies. It is worth to mention that despite the promising results reported for these technologies, significant variability is observed across studies. Such discrepancies may arise from differences in cultivar, geographical origin, ripening stage, pre-treatment conditions, solvent systems, extraction parameters, and analytical quantification methods. Therefore, caution is required when directly comparing reported values. Future research should prioritize methodological standardization and improved reporting of experimental parameters to enhance reproducibility and industrial scalability.

### Microwave-assisted extraction

Microwave-assisted extraction (MAE) involves heating a mixture of raw material with the solvent using microwave radiation energy (Alara & Abdurahman, 2019; Sánchez-Camargo et al., 2021). Increasing the temperature of the solute–solvent system accelerates the solvent penetration into the cells, which improves the diffusion of bioactive compounds from the fruit by-products into the solvent (Alara et al., 2018). This effect is attributed to improved solvent penetration into the matrix, which weakens the hydrogen-bond network within the sample and facilitates the transfer and solubilization of target compounds into the extracting medium (Shams et al., 2015). MAE is generally effective for recovering low-molecular-weight polyphenols, particularly phenolic acids and flavonoids; however, it is less commonly applied to polymeric polyphenols (*e.g*., anthocyanins and tannins) because microwave conditions may promote degradation of highly hydroxylated and heat-labile compounds, especially anthocyanins (Alara, Abdurahman & Ukaegbu, 2021).

Although the efficiency of microwaves in the extraction of phenolic compounds has been demonstrated, it is important to identify the appropriate extraction conditions, not only to increase the yield of phenolic compounds in the extracts, but also to evaluate how these conditions affect the concentration of the compounds of interest and their biological activities. In this sense, a Box-Behnken design has been used on multiple occasions to determine the optimal conditions for the microwave-assisted extraction process of phenolic compounds. Accordingly, it has been established that using microwaves (600 W), the best conditions for the extraction of antioxidant polyphenols (1,738.2 mg trolox equivalents (TE)/g; half maximal inhibitory concentration (IC_50_) of 0.078 mg/mL) from mango seeds kernel from cultivar Ataulfo are: 75 °C, a solid to solvent ratio of 1:60 g/mL (Torres-León et al., 2017). In the case of mango peel, Pal & Jadeja (2020) reported that using deep eutectic solvents with microwave-assisted extraction on the cultivar Kesar yielded extracts 56.17 mg gallic acid equivalents GAE/g of phenolic concentration and 683.27 µmol ascorbic equivalents/g of antioxidant activity were obtained when employing microwave power of 436.45 W, with a solid to liquid ratio of 59.82 mL/g and time process of 19.66 min (Sánchez-Camargo et al., 2021). Ramírez-Brewer, Quintana & García-Zapateiro (2024) reported an optimization of the microwaved extraction of phenolic content and antioxidant activity from mango peel of the Tommy Atkins and Sugar cultivars by using response surface methodology and artificial neural networks. For Tommy peel extract, 121.30 mg GAE/g and 1,185.9 µmol TE/g extract were obtained, while for the Sugar peel extract the maximum content were 224.86 mg GAE/g and 2,117.7 µmol TE/g extract.

In addition, Vélez-Erazo et al. (2021) optimized MAE for bioactive compounds such as flavonoids and carotenoids from mango peel by-products using response surface methodology, achieving good model fit for total phenolic content (TPC), total flavonoid content (TFC), and total carotenoid content (TCC). Although no clear optimal regions were observed, conditions could be tailored to obtain extracts enriched in polar compounds (675 W, 105 s, 50% ethanol; total extraction yield 62.80%, TPC 145.92 mg GAE/g dm, TFC 21.06 mg CE/g dm) or nonpolar compounds (675 W, 150 s, 80% ethanol; TCC 168.60 µg β-carotene/g dm). Additionally, kinetic modeling confirmed the suitability of low-cost MAE equipment, with all models showing strong agreement with experimental data (R^2^ > 91%). The two-site kinetic model provided the best fit (R^2^ > 95%), revealing that microwave irradiation accelerates both washing and slow diffusion phases, thus elucidating key mechanisms governing compound release during MAE.

MAE offers significant reductions in extraction time and solvent use. However, large-scale implementation remains technically challenging due to non-uniform microwave energy distribution in high-volume reactors, which may compromise reproducibility and process control. The relatively high cost of industrial microwave systems and the need for optimized reactor design can limit widespread adoption (Lama-Muñoz & Contreras, 2022; Wang et al., 2025) (Table 3).

**Table 3:** Comparative summary of main green extraction methods for the recovery of bioactive compounds.

Extraction technique	Operating principle	Key advantages	Limitations	Critical parameters	Relative energy demand	Industrial maturity	Scalability status	Main economic challenge
MAE	Volumetric microwave heating → rapid solvent/matrix heating, enhanced diffusion and cell disruption	Short extraction time; low solvent consumption	Requires careful temperature optimization; high temperature may cause degradation of thermally sensitive compounds	Solvents, ratio L/S, microwave power, temperature, extraction time.	Moderate to high (depends on the scale and reactor design)	Pilot to semi-industrial scale	Limited large-scaleimplementation; scale-up still under optimization	High equipment cost; scale-up limitations due to non-uniform microwave distribution
UAE	Acoustic cavitation by ultrasonic waves → enhanced mass transfer	Easy to execute; simple; economical equipment; low solvent consumption; rapid extraction; good extraction yield with high reproducibility; low impact to the environment. Compatible with green solvents	High ultrasound waves degrade bioactive compounds due to oxidative pyrolysis caused by hydroxyl (OH-) radicals during cavitation phenomenon	Solvents, ratio L/S, extraction time, amplitude, frequency and intensity	Moderate	Pilot to industrial (in selectedapplications)	Good scalability potential with flow-through systems	Reactor design complexity; efficiency loss at large volumes
PLE	Pressure with high temperature → lower viscosity/higher diffusivity increase solubility	Consumes less organic solvents; fast and efficient; possibility to avoid organic solvents by using water only	Extraction temperature must be optimized; high temperatures may lower recovery of thermolabile polyphenols. Expensive instrumentation	Solvents, ratio L/S, temperature, pressure and extraction time	Moderate to high	Primarily laboratory to pilot scale	Limited industrial adoption; more common in analytical applications	High-pressure equipment cost; solvent recovery systems required
SFE	Supercritical fluids solvating power tunable with P/T; modifiers increase polarity	High selectivity; safer and green solvent; easily controlled extraction conditions; low operating temperature; environmental friendliness; easy separation of solvents from solutes	Low total yield for very polar active compounds. High pressure equipment cost	Pressure, temperature, co-solvents, solvent flow rate, time.	High (due tocompression requirements)	Industriallyimplemented in some sectors (*e.g*., specialty extracts)	Industrially scalable but capital-intensive	Very high capital investment; high-pressure operation costs
PEF and HVED	Electroporation → increased membrane permeability, enhanced mass transfer	Non-thermal; fast; can cut solvent and time; improves mass transfer/selective release	Potential formation of reactive species; need control to avoid compound alteration; scale-up less mature	kV-range discharges in liquid/solid-liquid systems; highly setup-dependent	Moderate to high	PEF: Industrially implemented in some sectors HVED: Emerging/experimental	PEF: Good scalability potential. HVED: Currently limited to pilot scale	Specialized equipment; process control challenges; limited commercial data
EAE	Enzymatic hydrolysis of cell wall polysaccharides → release bound compounds	Safe and green; High selectivity; mild conditions.	Long extraction time; low-efficiency; expensive	Type and concentration of enzyme, temperature, pH, solvents, extraction time, and substrate concentration	Low to moderate	Industrially feasible	Highly scalable; compatible with conventional processing	Cost of enzymes; process time; enzyme recovery and reuse

**Note:**

Sources: Ameer, Shahbaz & Kwon (2017), Gil-Martín et al. (2022), Lama-Muñoz & Contreras (2022), Zhang et al. (2022), Tan (2025), Wang et al. (2025).

### Ultrasound-assisted extraction

Ultrasound-assisted extraction (UAE) is another green extraction technique that has demonstrated to be highly efficient in the extraction of bioactive compounds from plant raw materials (Wang, Li & Bi, 2018). This method exploits the phenomenon of acoustic cavitation, which is produced when ultrasonic waves create rapid fluctuations in temperature and pressure, causing the collapse of microbubbles in the medium (Chemat et al., 2017; Tacias-Pascacio et al., 2022). These violent implosions fragment or disrupt the surface of the solid matrix, thereby enhancing mass transfer and accelerating the diffusion process (Chemat et al., 2017). Moreover, the formation of pores in cell membranes during cavitation, is a phenomenon known as sonoporation, this facilitates the release of intracellular bioactive compounds (Azmir et al., 2013; Corbin et al., 2015; Kumar, Srivastav & Sharanagat, 2021). Ultrasound also increases the water absorption of the pomace, improving the solvent’s accessibility to the bioactive compounds and boosting their diffusivity (Pingret et al., 2012). In addition, UAE elevates swelling index of the plant tissue matrix aids both desorption and diffusion of solutes, leading to an increased extraction efficiency (Dezhkunov & Leighton, 2004; Chemat et al., 2017).

The effectiveness of UAE depends on several parameters such as amplitude (% or µm), frequency (Hz), intensity (W/cm^2^), power (W) and speed (m/s) which must be carefully optimized (Kumar, Srivastav & Sharanagat, 2021). Generally, UAE can be implemented either directly in the system (solid-liquid extraction) or indirectly, utilizing a piezoelectric transducer as the ultrasound source (Tacias-Pascacio et al., 2022). In direct sonication, an ultrasonic probe (or horn) connected to a transducer is immersed in the extraction medium, delivering ultrasound waves directly with minimal energy loss. In the case of indirect sonication is performed in an ultrasonic bath, where the solid matrix is dispersed in the solvent in a stainless-steel tank connected to the transducer; here, the sample is contained in a separate vessel, and the ultrasound waves pass through the walls (Wen et al., 2018). Although ultrasonic baths are more economical and easier to handle, their low reproducibility restricts its use in extraction process compared to the direct method (Kumar, Srivastav & Sharanagat, 2021; Tacias-Pascacio et al., 2022). The direct probe-based systems are commonly preferred because they concentrate higher ultrasonic intensity in a specific zone, which results in a more efficient cavitation effect (Adetunji et al., 2017; Chemat et al., 2017; Kumar, Srivastav & Sharanagat, 2021).

An optimal UAE extraction process requires an experimental design such as the response surface methodology. Ojeda et al. (2022) optimized ultrasound-assisted extraction conditions employing response surface methodology using three-factors (% ethanol in water, amplitude and time) to maximize the recovery of antioxidant compounds such as total phenolic content (TPC), total flavonoid content (TFC) and antioxidant activity (ABTS and DPPH assays) in different sections of the fruit (peel, pulp and seeds) of the underutilized mango criollo. The operational optimum conditions to maximize extraction were different for each part of the mango: peel, 46% ethanol/amplitude 60 µm/time 6.5 min; pulp, 25% ethanol/amplitude 45 µm/time 30 min; seeds, 49% ethanol/amplitude 60 µm/time 20 min. These results proved that the use of experimental design is a key step in order to maximize the extraction procedure in different fruit sections. In this study UAE increased up to 33% the extraction yield of phenolic compounds compared to a conventional extraction.

One of the most critical factors in the optimization of ultrasound-assisted extraction is the choice of solvent. The physicochemical properties of the solvent such as polarity, vapor pressure, surface tension, and viscosity can influence the wave of propagation, consequently affecting the extraction efficiency (Sim, Ong & Nyam, 2019; Martínez-Ramos et al., 2020; Herrera-Pool et al., 2021). For instance, when extracting phenolic compounds from mango peel using a sonication bath (35 kHz) and 80% ethanol, a high phenolic content 67.58 mg GAE/g was obtained (Safdar, Kausar & Nadeem, 2017). In contrast, employing an ultrasonication probe with an ethanol-acetone mixture increased the yield of phenolic compounds extracted by up to 630%, however decreased the antioxidant activity because it could favour the generation of sonochemical reactions such as polymerization or degradation (Martínez-Ramos et al., 2020; Monteiro et al., 2020; Tacias-Pascacio et al., 2022). In another study, with a lower sonication power (20 kHz), the optimal extraction of phenolic compounds from mango peel of Tommy Atkins was obtained using a 50% ethanol solution (Guandalini, Rodrigues & Marczak, 2019). Furthermore, Lanjekar, Gokhale & Rathod (2022) explored the combination of ultrasound with natural deep eutectic solvents (NADES) to reduce extraction time and solvent consumption. Under optimized conditions, 20% water content, 50% duty cycle, 2 W/cm^3^ acoustic density, a 30:1 liquid:solid ratio, 0.3 mm particle size, and a 30 min extraction time the lactic acid-glucose NADES (5:1) achieved a TPC of 69.85 mg GAE/g, a total flavonoid content (TFC) of 16.5 mg QE/g and a DPPH IC_50_ of 35.37 µg/mL. Compared to a bath process with 80% ethanol, the UA-NADES technique yielded 1.4 times higher TPC, 1.7 times higher TFC, and 1.9 times greater antioxidant activity, while reducing extraction time by 50% and solvent consumption by 25%.

UAE has proven to be effective for carotenoid recovery from different food processing by-products (Linares & Rojas, 2022). For instance, Ghoshal, Singh & Sharma (2022) reported that sonication during the preparation of flaxseed oil-enriched mango significantly enhanced β-carotene content, increasing from 151.37 to 292.24 µg/mL as oil concentration rose from 0% to 0.75%. This improvement was attributed to ultrasound-induced cell disruption, which facilitates the release of carotenoids and other lipophilic compounds. Also, Rodríguez-Rodríguez et al. (2024), has demonstrated that the combination of UAE with green solvents (2-metiltetrahydrofuran and cyclopentyl methyl ether) was a suitable and sustainable procedure for recovery carotenoids from mango pulps.

UAE demonstrates strong scalability potential, particularly in continuous flow systems. Compared to conventional extraction, UAE may reduce processing time and solvent consumption, contributing to operational efficiency. Nevertheless, acoustic energy distribution and equipment design must be carefully optimized at industrial scale to maintain extraction performance. Economic feasibility depends largely on system configuration and process integration (Ameer, Shahbaz & Kwon, 2017; Lama-Muñoz & Contreras, 2022; Tan, 2025; Zhang et al., 2022) (Table 3).

### Pressurized liquid extraction

Pressurized liquid extraction (PLE), also termed accelerated solvent extraction, is a pressurized high-temperature solid–liquid extraction technique designed to improve the recovery of phytochemicals by intensifying mass-transfer phenomena. In PLE, temperatures typically between 40 °C and 200 °C and pressures around 3.3–20.3 MPa are applied simultaneously to keep the solvent in the liquid phase while modifying its physicochemical properties (*e.g*., viscosity, diffusivity, surface tension, and solvating power), thereby enhancing analyte desorption from the matrix and increasing solubility in the extracting medium (Nieto et al., 2010; Zhang, Lin & Ye, 2018; Alara, Abdurahman & Ukaegbu, 2021). Relative to conventional extraction, these conditions can accelerate extraction kinetics and reduce solvent requirements while maintaining efficient analyte transfer (Nieto et al., 2010). A key advantage is the feasibility of using water under subcritical conditions as an extraction solvent (subcritical water extraction), where elevated temperature shifts water’s dielectric behavior and reduces its polarity, allowing it to act more similarly to organic solvents and thus broaden the range of extractable compounds (Plaza & Turner, 2015; Teo et al., 2010; Alara, Abdurahman & Ukaegbu, 2021). PLE can also produce comparatively “cleaner” extracts than some traditional approaches, potentially minimizing extensive downstream clean-up prior to high-sensitivity analyses such as LC–MS (Sosa-Ferrera, Mahugo-Santana & Santana-Rodríguez, 2013; Alara, Abdurahman & Ukaegbu, 2021). Nevertheless, PLE may exhibit limited selectivity due to co-extraction of matrix components, can yield diluted extracts when multiple static/dynamic cycles are applied, and requires specialized high-pressure instrumentation, which increases operational costs. Despite these constraints, PLE has been widely applied for polyphenol recovery from diverse sources (Erdogan et al., 2011; Liazid et al., 2014; Alara, Abdurahman & Ukaegbu, 2021). Moreover, PLE has demonstrated high efficiency for recovering lipophilic compounds such as carotenoids in other plant matrices such as carrots or pressed palm fiber (Cardenas-Toro et al., 2015; Šaponjac et al., 2021). Although no studies have reported carotenoid extraction from mango using PLE, its proven performance in similar substrates suggests strong potential for this application, warranting further research to optimize conditions for mango peel and pulp.

Optimizing PLE performance involves adjusting several operational variables, with solvent selection being among the most influential factors. Although the elevated temperatures used in PLE modify solvent physicochemical properties (and can therefore shift extraction behaviour), overall efficiency and selectivity still depend strongly on the chosen solvent system. In practice, phenolic compounds are frequently extracted with methanol or ethanol, often in aqueous mixtures at different proportions to tune solvent polarity and matrix penetration. Because phenolics encompass structurally diverse subclasses, solvent composition should be selected systematically, as different phenolic families can respond very differently to the same extraction conditions within a single sample (Alara, Abdurahman & Ukaegbu, 2021). In the context of mango matrices, these considerations are particularly relevant, since available evidence although still limited compared with other techniques suggests that PLE can be tailored to recover targeted phenolic fractions with competitive performance.

Although not a widely used method for extracting phenolic compounds from mango fruit, PLE has shown notable promise. For instance, Sánchez-Mesa et al. (2019) found that while PLE yielded a higher overall recovery of phenolic compounds from mango peel compared with supercritical fluid extraction (SFE), SFE in general provided a higher concentration of compounds (gallotannins, xanthones, flavonoids, *etc*.), with gallic acid being the main outlier, likely due to its greater affinity for pressurized water, which enhanced its recovery by PLE. The optimal PLE conditions in this study were 6.67 g/min of Mili-Q water at 40 °C and 10 MPa. In contrast, the best SFE conditions were achieved at 50 °C, 20 MPa and co-solvent flow rate corresponding to 20% of ethanol with CO_2_.

In case of mango seed kernel, Ballesteros-Vivas et al. (2019) implemented a recovery with PLE in two step process: first isolating the non-polar fraction (fatty acids and lipids) and then the polar fraction (polyphenols). The success for the extraction depended on the selection of the most suitable solvent for the second PLE step, with a mixture of ethanol/ethyl acetate. Following the extraction, they obtained a phytochemical profiling by GC- and LC-Q-TOF-MS/MS, fully characterizing the fractions obtained. Furthermore, the mango seed kernel-extract produced by PLE was evaluated for its the antiproliferative activity against colon cancer cells. Compared to conventional extraction techniques (*e.g*., Soxhlet), the PLE-derived extracts showed significantly higher inhibition of proliferation in the HT-29 human colon adenocarcinoma cell line. These findings demonstrate the potential of the proposed valorization strategy to offer the mango processing industry to produce a value-added product to the market with health promoting properties (Ballesteros-Vivas et al., 2019).

Additional studies have demonstrated that PLE outperforms SFE in the extraction of compounds from mango leaves. While SFE extracts of mango leaves is mainly limited by diffusion, PLE extracts are controlled by both convection and diffusion, leading to more efficient extraction (Fernández-Ponce et al., 2016). In another investigation, researchers extracted with PLE using ethanol phenolic compounds from various plant leaves, and evaluated the phenolic content and antioxidant activity. They found out that low concentrations of ethanolic extracts improved angiogenic capacities of endothelial colony forming cells (ECFCs) and also reduced their proliferation, apoptosis, and inflammatory response. Overall, although the ethanolic extracts from mango leaves provided the most promising results, all the three evaluated extracts contributed to improve the functionality of ECFCs (Sánchez-Gomar et al., 2022).

PLE is widely used in laboratory and analytical contexts due to its high extraction efficiency under controlled temperature and pressure conditions. However, the requirement for high-pressure equipment, solvent recovery systems, and safety controls increases capital and operational costs. As a result, PLE remains less common in large-scale food processing applications (Lama-Muñoz & Contreras, 2022; Tan, 2025) (Table 3).

### Supercritical fluid extraction

Supercritical fluids represent a sustainable alternative to conventional organic solvents used in extraction processes. A fluid reaches its critical state when it is heated above its critical temperature (Tc) and pressurized beyond its critical pressure (Pc) (Brunner, 2005). In this state, the fluid simultaneously exhibits a unique blend of properties: it maintains a density similar to that of liquids providing a solvating power similar to liquids, while its viscosity remains close to that of gases and its diffusivity remains intermediate between that of liquids and gases. This unique combination of properties improves mass transfer and enables the fine-tuning of solvent selectivity toward specific target compounds, thereby optimizing the extraction process (Chemat et al., 2017). Moreover, extraction using compressed supercritical fluids has the great advantage to be innocuous to food components and safe for human consumption, while also minimizing environmental impact by eliminating toxic solvents and reducing the high energy demands of alternative extraction methods (Brunner, 2005).

Supercritical CO_2_ (SC-CO_2_) is the most widely used fluid in SFE of natural matrices (Rozzi & Singh, 2002; Da Silva, Rocha-Santos & Duarte, 2016). Its application is facilitated by its relatively low critical conditions (Tc: 31.60 °C, Pc: 7.38 MPa). Additionally, offers some advantages: it is non-toxic, non-flammable, non-corrosive, thermodynamically stable, chemically inert, and non-mutagenic, while its abundance and volatility at atmospheric pressure ensure solvent-free extracts after a depressurization (Wrona et al., 2017; Zhou et al., 2021; Gil-Martín et al., 2022). As a non-polar solvent with solvating power intermediate between pentane and toluene, SC-CO_2_ permits the use of various pressure-temperature combinations, thereby making it an ideal technique for producing multiple end products (Chemat et al., 2017; Alara, Abdurahman & Ukaegbu, 2021). This versatility not only avoids the use of organic solvents but also prevents the degradation of thermolabile compounds in lab-scale applications. Furthermore, its low operating temperatures and pressures preserve thermolabile phytochemicals, and benefits such as higher mass transfer due to decreased viscosity and increased diffusion, enhanced solvent penetration, adjustable extraction conditions, and recyclability further attest its effectiveness and environmental compatibility (Alara, Abdurahman & Ukaegbu, 2021; Gil-Martín et al., 2022).

Despite its advantages, SC-CO_2_ is limited by its low polarity, which favors the extraction of non-polar compounds and reduces performance for highly polar molecules such as polyphenols bearing hydroxyl and carboxyl groups (Fiori et al., 2009). This limitation can be mitigated by increasing pressure (higher density) to improve extraction of targets like flavonoids (Bimakr et al., 2011) and/or by adding 1–10% polar co-solvents (*e.g*., ethanol, water, ethyl lactate) to promote recovery of polar compounds, including polyphenols (Khaw et al., 2017; Rodríguez De Luna, Ramírez-Garza & Serna Saldívar, 2020; Gil-Martín et al., 2022). These cosolvents, or modifiers undergo dipole-dipole and hydrogen-bonding interactions that enhance the solubility of polar compounds at temperatures suitable for thermolabile species (Cvjetko Bubalo et al., 2018; Gil-Martín et al., 2022). Due to its low toxicity, ethanol is the preferred option for nutraceutical and food applications, especially considering that pure water is highly corrosive in the supercritical state (critical point: 374 °C, 22 MPa) and hence unsuitable as cosolvent for polyphenol extraction (Dai & Mumper, 2010). Nevertheless, the high pressures required in SFE require expertise and specialized equipment such as pressurization unit, gas storage and pressure sample vessel, among others, which may compromise accessibility and makes scalability expensive (Panja, 2018).

In case for the extraction of mango by-products Cerón-Martínez et al. (2021) optimized the extraction of oil rich in essential fatty acids from mango seed kernels using a pilot-scale SC-CO_2_ plant. They reported a maximum oil yield of 83 g/kg at 37 MPa and 63 °C, while response surface methodology predicted 84 g/kg at 35 MPa and 65 °C. By fine-tuning the conditions, an EFA-rich lipid fraction was obtained with 37 g/kg linoleic acid, 4 g/kg α-linolenic acid, and 155 g/kg oleic acid at 23 MPa.

Similarly, Villacís-Chiriboga et al. (2021) extracted bioactive compounds from mango peel and pulp, using response surface methodology to model carotenoid recovery. Optimal conditions were determined to be 55 °C, 35 MPa, and 20% ethanol, with β-carotene as the predominant carotenoid and the peel showing up to 4.1 times higher bioactive content than the pulp. Furthermore, Bello et al. (2024) investigated the extraction of bioactive compounds from mango peel waste under supercritical CO_2_ conditions in order to evaluate its potential for oxidative stabilization of biodiesel. The experimental setup included a CO_2_ flow rate of 9.8 g/min, a fixed pressure of 25 MPa, and temperatures ranging of 40 °C to 80 °C, over durations of 30 to 150 min. High-performance liquid chromatography (HPLC) was employed to isolate quercetin, β-carotene and gallic acid, whose quantities were estimated to be 1.2983, 3.6987, and 0.0254 mg/g, respectively (Bello et al., 2024). In a similar approach, Baysal, Ersus & Starmans (2000) optimized supercritical CO_2_ extraction of lycopene and β-carotene from tomato paste waste using a factorial design. They reported that the highest lycopene yield (53.93%) was achieved at 55 °C and 300 bar with a CO_2_ flow rate of 4 kg/h and 5% ethanol as a cosolvent, while β-carotene recovery was maximized at 65 °C under the same pressure and cosolvent conditions (Baysal, Ersus & Starmans, 2000).

SFE, particularly when using CO₂, has already reached industrial maturity in certain high-value extract markets. Its advantages include solvent-free extracts and selective compound recovery. However, the technology is capital-intensive, requiring high-pressure equipment and significant energy input for compression. These economic factors may restrict its application to products with sufficient added value (Lama-Muñoz & Contreras, 2022; Tan, 2025) (Table 3).

### Electrical force methods: pulsed electric fields and high voltage electrical discharges

Nonthermal electrochemistry entails applying electric fields under various regimes to impose physical stress on plant cell walls and membranes, markedly enhancing their permeability to solvents so that intracellular phytochemicals become extractable. This process, also known as electroporation, refers to the increase in cell membrane permeability induced by an external electric field. In this context, two prominent techniques can be included: pulsed electric fields (PEF) and high voltage electrical discharges (HVED). The goal of nonthermal sample electrostimulation is to improve mass transfer, thereby reducing the requirements for energy, time and solvents. Recently, several electro-technical modalities fully aligned with the green chemistry principles have been developed for valorization of plant wastes and by-products (Vorobiev & Lebovka, 2010; Gil-Martín et al., 2022).

PEF extraction is based on exposing the vegetal matrix to a controlled electrical potential. A transformer generates electric pulses, with voltages ranging from 140 or 220 V up to 1,000 V or even surpassing 25,000 V that are discharged into a sealed chamber containing the sample for durations from a few microseconds to several hundred seconds (Rodríguez De Luna, Ramírez-Garza & Serna Saldívar, 2020; Gil-Martín et al., 2022). These electric pulses stress the cell membrane, inducing the formation of pores that may be reversible or irreversible depending on the intensity of the treatment: electric field strength, energy applied, shape and duration of the pulse, and number of pulses (Gil-Martín et al., 2022).

PEF (typically 0.5–10 kV/cm) treatment accelerates the extraction of plant bioactive compounds by inducing electroporation, a process that increases cell membrane permeability, enhances electrical conductivity, and accelerates mass transfer (Vorobiev & Lebovka, 2010; Boussetta, Lesaint & Vorobiev, 2013; Gil-Martín et al., 2022). Generally, the electric field/mass ratio is the main factor for optimizing PEF extractions, since a greater electric pulse leads to a larger pore sizes and increased the disintegration of cell walls and membranes (Peiró et al., 2019; Rodríguez De Luna, Ramírez-Garza & Serna Saldívar, 2020).

The effectiveness of this technique relies in its ability to disrupt the plant cell membrane of the matrix. It is important to note that pulsed electric fields, in addition to produce electroporation of cell membranes, may stimulate the biosynthesis of secondary metabolism as a self-protective response, which has been associated with an increase in phenolic and carotenoid compounds and other secondary metabolites, as it has been observed in certain matrices such as whole tomatoes (Soliva-Fortuny et al., 2009). Therefore, the intensity of PEF protocols must be carefully modulated to achieve an optimal balance between energy consumption and extraction efficiency of native compounds.

Furthermore, the combination of PEF with solvent extraction has demonstrated promising results in the recovery of phenolics through the modulation of temperature and pH conditions. Improvements in solvent consumption, extraction time, and yield compared to conventional liquid partitioning have been reported for the valorization of plant by products such as mango peel (Parniakov et al., 2016). In addition, it has been observed that temperature has a negligible impact on the aqueous extraction of proteins and carbohydrates, while a two-stage procedure, involving initial PEF-assisted extraction followed by supplementary aqueous extraction at 50 °C with adjusted pH over 3 h, yielded a significant (~400%) enhancement in TPC recovery even at neutral pH. This clearly demonstrates that pulsed electric field-assisted extraction is an effective technology for the selective extraction of bioactive compounds.

HVED is another nonthermal extraction method and is considered a plausible alternative to conventional extraction techniques (Li, Fan & Xi, 2019). Although its operating principle is similar to that of PEF, HVED differs in that the discharges occur at a localized point. In HVED, the solvent–solid mixture is placed in a chamber that is sealed with a lid, ensuring that an electrode is in direct contact with the mixture. The intense electric fields from 20 to 40 kV are applied through two electrodes propagate through the solvent to the sample inside the discharge chamber, and above certain potential limit is exceeded the electroporation of cell wall and membrane is produced (Rodríguez De Luna, Ramírez-Garza & Serna Saldívar, 2020; Gil-Martín et al., 2022). The electroporation, similar to that induced by PEF, increases the diffusivity of intracellular components and reduces extraction times.

However, the formation of high-energy streamers and arcs leads to electrochemical and chemical reactions such as the generation of reactive radicals and hydrogen peroxide or ozone, which have not yet been fully characterized. These reactions must be further addressed in order to understand their impact on the chemical integrity of target species and viability of subsequent biotransformation processes (Saulis et al., 2015; Gil-Martín et al., 2022). Consequently, it is essential to establish the highest voltage that enhances the solubilization of targeted phenolics without exceeding a limit that could induce electro-related adulteration (Nutrizio et al., 2020; Rodríguez De Luna, Ramírez-Garza & Serna Saldívar, 2020; Gil-Martín et al., 2022).

The distance between the electrode and the discharge plate also significantly influences extraction performance. In general, very large distances weaken the electric field intensity, whereas excessively short distances reduce the duration of the electric arc, diminishing the energy impact on the plant material. For this reason, an optimal distance must be determined, although this will depend mainly on the type of solvent (Boussetta et al., 2011; Rodríguez De Luna, Ramírez-Garza & Serna Saldívar, 2020). Another influential factor is the solvent volume (liquid–solid ratio), which partly governs the diffusion rate of soluble matrix components. Initially, as solvent availability increases, the extraction rate rises; however, beyond a certain point, further improvement levels off into a dynamic plateau equilibrium (Rodríguez De Luna, Ramírez-Garza & Serna Saldívar, 2020). Moreover, HVED efficiency is affected by other operational factors, such as treatment time and the choice of extraction solvent. For example, in the extraction of flavan-3-ols, flavonols, and stilbenes from grape stems, treatment time, pH, and ethanol concentration significantly affected extractability especially for flavan-3-ols and flavonols (Brianceau et al., 2016; Gil-Martín et al., 2022). It is important to note that both PEF and HVED have demonstrated significant yield improvements along with reductions in time and temperature as observed in the extraction of polyphenols from mango peel (Parniakov et al., 2016). Building on these findings, Santos et al. (2023) explored the application of PEF as a non-thermal pre-treatment to enhance the dying efficiency and functional quality of mango peel. By inducing electroporation, PEF improved mass transfer across cell membranes, facilitating moisture removal and compound retention. The study assessed the influence of PEF intensity at different drying temperatures, with the most effective condition observed at 4.5 kV/cm combined with drying at 70 °C. Under these parameters, drying time was reduced by up to 67%, while degradation of total phenolic content was minimized to 24.30%. Additionally, this condition yielded higher solubility (78.48%) and glass transition temperature (59.97 °C). Antioxidant activity, measured *via* the DPPH method, reached peak values (69.27 and 176.21 µmol TE/g), and digestibility after 120 min was 26.04%. Additionally, PEF has been applied beyond extraction purposes, notably in postharvest preservation strategies. Supasin et al. (2022) evaluated the combination of PEF and chemical pickling in *Chok-Anan* mangoes, reporting extended shelf-life and improved retention of quality attributes, including color. Interestingly, the PEF-assisted pickling process resulted in a 20% increase in β-carotene content but a 47% reduction in ascorbic acid. The loss of vitamin C was attributed to accelerated leaching into the osmotic solution and possible oxidation reactions, as PEF can disrupt hydroxyl groups and alter molecular configuration. Although antioxidant activity decreased due to ascorbic acid degradation, this effect was partially offset by the rise in β-carotene, which also exhibits antioxidant properties. Structural modifications induced by electroporation further contributed to these changes. Overall, these findings reinforce the potential of non-thermal technologies such as PEF as effective pre-treatments to enhance the functional quality and preservation of agro-industrial products.

The PEF-assisted extraction technique requires moderate to high pulsed electric fields (0.5–10 kV/cm), low energy consumption, low solvent, and short processing times, while providing higher extraction yields of different bioactive compounds from fruits, vegetables, spices, leaves, and their wastes or by-products. However, the large-scale industrialization of this process currently requires the design of larger treatment chambers.

HVED is considered an emerging technology with promising extraction enhancement capabilities. Nevertheless, its industrial adoption is still limited due to the need for specialized equipment, process control challenges, and insufficient large-scale validation data. Further pilot-scale studies are required to assess its technoeconomic feasibility (Gil-Martín et al., 2022) (Table 3).

### Enzyme-assisted extraction

Enzyme-assisted extraction (EAE) is a method that leverages the biocatalytic activity of specific enzymes to break down plant cell walls and release intracellular bioactive compounds (Nadar, Rao & Rathod, 2018). Plant cell walls are complex matrices composed of polysaccharide networks such as cellulose, hemicellulose, and pectin along with lignin and proteins, which confer structural stability and resistance against extraction (Sowbhagya & Chitra, 2010; Rodríguez De Luna, Ramírez-Garza & Serna Saldívar, 2020). In the EAE process, enzymes (including pectinases, cellulases, and hemicellulases) bind specifically to their substrates in the cell wall and catalyze the hydrolysis of bonds. This enzyme-substrate interaction induces conformational changes that ultimately disrupt the integrity of the cell wall, increasing its permeability and allowing the release of compounds such as oils, polyphenols, polysaccharides, pigments, and other medicinal substances (Nadar, Rao & Rathod, 2018; Rodríguez De Luna, Ramírez-Garza & Serna Saldívar, 2020).

Many plants phenolic compounds are tightly integrated within the wall architecture. They can be retained by hydrogen and hydrophobic bonds within the polysaccharide–lignin matrix, form ether-type bonds with lignin, or remain esterified to carbohydrates and proteins (Rodríguez De Luna, Ramírez-Garza & Serna Saldívar, 2020). Consequently, pretreatment with degrading enzymes (or a combination of pectinolytic and polysaccharide-degrading enzymes) disrupts these interactions. This disruption enhances solvent accessibility, improves solvation and mass transfer, and facilitates the extraction of phenolics that often remain inaccessible to conventional solvents (Papillo et al., 2014; Nadar, Rao & Rathod, 2018; Gil-Martín et al., 2022).

EAE has proven particularly efficient for extracting polyphenols, notably in recovering anthocyanidins and flavonoids from their glycosidic forms (Gligor et al., 2019). While some phytochemicals are readily dispersed in the plant cytoplasm, others remain sequestered within the complex polysaccharide–lignin network and are less accessible by traditional extraction methods (Rodríguez De Luna, Ramírez-Garza & Serna Saldívar, 2020). One of the key advantages of enzymatic extraction is its environmentally friendly nature: the process typically operates in water, under mild conditions and for short periods, thereby offering high substrate specificity while reducing the need for hazardous organic solvents (Gligor et al., 2019; Gil-Martín et al., 2022).

Nevertheless, the efficiency of EAE is highly dependent on several physicochemical parameters, such as the composition of the enzyme mixture, pH, temperature, and particle size. Most EAE protocols are conducted under acidic conditions, as low pH values favor the breakdown of the secondary bonds that secure phenolic compounds to cell wall components (Gil-Martín et al., 2022). Moreover, reducing particle size increases enzyme accessibility to the susceptible bonds and shortens the diffusion paths for the released bioactives, further optimizing the extraction yield (Reverchon & De Marco, 2006; Gil-Martín et al., 2022). Some drawbacks are the high cost of the enzymes and the scale-up of EAE process can be difficult because of the uniqueness in response of different enzymes to changing environmental conditions (Wongkaew et al., 2021a).

James et al. (2025) investigated the effects of various extraction techniques and processing conditions on the recovery of pectin and antioxidants from mango peels. Antioxidant analysis revealed that both EAE and UAE yielded the highest total phenolic content and exhibited the greatest antioxidant activity, with DPPH radical scavenging reaching 84%. For the EAE method, 10 grams of freeze-dried, ground mango peel were suspended in 300 mL of 60% methanol. The sample was then immersed in distilled water, treated with 3% alpha-amylase, and incubated at 70 °C for 30 min. Although recent studies on the use of EAE for extracting bioactive compounds from mango are limited, this technique has been applied for the extraction of pectin and dietary fiber from mango peels and seeds (Viscusi et al., 2024; James et al., 2025). However, no reports exist on the enzymatic-assisted extraction of carotenoids from mango. Evidence from other matrices suggests its potential: Nath et al. (2016) optimized carotenoid recovery from sweet peppers using liquefaction enzymes such as viscozyme L and pectinase, achieving up to 87% extraction efficiency and a 2.3-fold increase compared to conventional n-hexane extraction. These findings highlight the feasibility of EAE for carotenoid recovery in mango by-products, warranting further investigation.

EAE is among the most easily scalable approaches, as it can be integrated into conventional processing systems with relatively moderate equipment modifications. Its main economic limitation lies in enzyme cost and potential issues related to enzyme stability, reuse, and process time. However, when properly optimized, EAE can offer a cost-effective and energy-efficient alternative for mango by-product valorization (Gil-Martín et al., 2022; Zhang et al., 2022) (Table 3).

To sum up, these emerging extraction technologies, MAE, UAE, PLE, SFE, HVED, and EAE are frequently characterized as sustainable alternatives to conventional methods due to improved efficiency and reduced solvent use. However, their industrial implementation requires evaluation beyond laboratory performance, considering capital expenditure, operational costs, energy consumption, and scalability constraints. MAE and UAE offer shorter processing times and enhanced mass transfer, but scale-up challenges may influence cost-effectiveness at industrial scale. PLE and SFE rely on high-pressure systems, resulting in increased equipment costs and energy requirements. While SFE has achieved commercial application in high-value extract production, its economic feasibility depends on product added value. PLE remains largely confined to analytical or pilot-scale operations. HVED represents an innovative approach with promising extraction enhancement capacity, yet its industrial maturity is still limited due to insufficient large-scale validation. In contrast, EAE is comparatively adaptable to conventional processing lines, although enzyme costs, stability, and reuse strategies must be optimized to ensure economic viability. It is clear, that while some techniques show strong potential at laboratory scale, further technoeconomic and life-cycle assessments are necessary to confirm their sustainability at industrial scale. Importantly, when these technologies are intended for the production of functional beverages enriched with mango-derived bioactive compounds, scalability and cost-efficiency become critical. Industrial beverage production requires consistent quality, regulatory compliance, and economically sustainable processes. Therefore, the successful translation of these green technologies into beverage formulations depends not only on extraction efficiency but also on technoeconomic optimization, process integration, and regulatory alignment.

## Major biological activities of mango products

Recent research on bioactive compound in mango has employed a variety of experimental models from *in vitro* cell studies to *in vivo* animal models and human trials to explore their diverse health-promoting effects. Studies indicate that compounds derived from both mango pulp and its by-products exhibit significant antibacterial, antioxidant, antidiabetic, anti-inflammatory, and anticancer activities (Gupta et al., 2022; Fong-in et al., 2025). By investigating the specific actions of these phytochemicals, researchers are gradually uncovering the mechanisms behind mango’s therapeutic potential. Notably, mango peel which account for 15–20% of the fresh fruit and is typically discarded during industrial processing is an abundant source of bioactive ingredients such as flavonol O- and xanthone C-glycosides, gallotannins, and benzophenone derivatives, with mangiferin standing out as a key phenolic compound due to its wide-ranging pharmacological properties (Imran et al., 2017; Fong-in et al., 2025). In addition, other compounds such as kaempferol, vanillic acid, and quercetin found in pulp, peel, and seed kernel have been reported to exert multiple biological activities, including antioxidant, anticancer, anti-inflammatory, and antidiabetic effects (Table 4). The following sections provide an in-depth discussion of their antioxidant, antidiabetic, anticancer, and anti-inflammatory properties.

**Table 4:** Biological activities of bioactive compounds isolated from mango.

Compound	Chemical structure	Fruit part isolated	Activities	References
Kaempferol	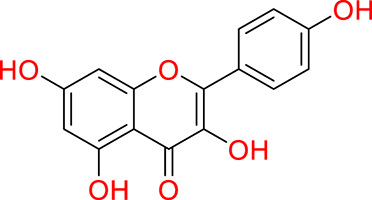	Pulp Peel Seed	Antioxidant activity, anti-cancer, anti-inflammatory, antidiabetic, neuroprotective	Lee et al. (2021), Choudhary et al. (2023), Parveen, Bhat & Bhat (2023)
Vanillic acid	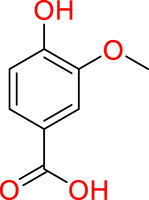	Pulp Peel	Antioxidant, antidiabetic, anti-inflammatory, anti-hyperinsulinemic	Alañón et al. (2021b), Zhao & Yang (2022), Ashokkumar & Vinothiya (2023), Duarte et al. (2025)
Quercetin	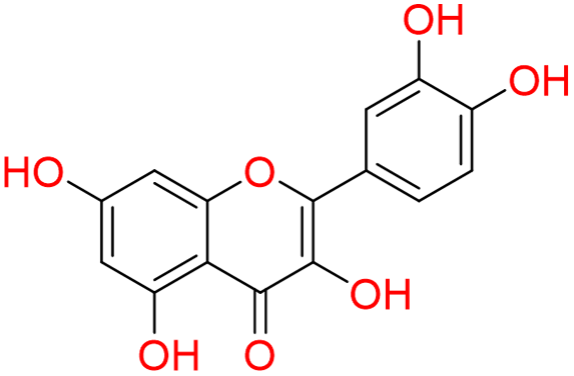	Peel	Antioxidant, antidiabetic, anti-bacterial, anti-inflammatory, anticancer	Mahadevan & Karwe (2016), Shabir et al. (2022), Shahana & Madheswaran (2024)
Gallic acid	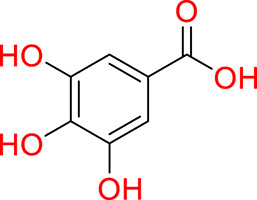	Pulp Peel Seed	Anti-inflammatory, antioxidant, antidiabetic, anticarcinogenic	Nag & Majumder (2023), Duarte et al. (2025)
β-Carotene	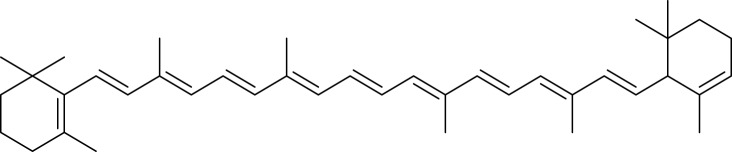	Peel Pulp	Antioxidant, anticancer, anti-inflammatory, antidiabetic, neuroprotective	Sánchez-Camargo et al. (2019), Marcelino et al. (2020), Cheng et al. (2021), Althurwi et al. (2022), Kabir et al. (2022), Kordiak et al. (2022)
α-Tocopherol	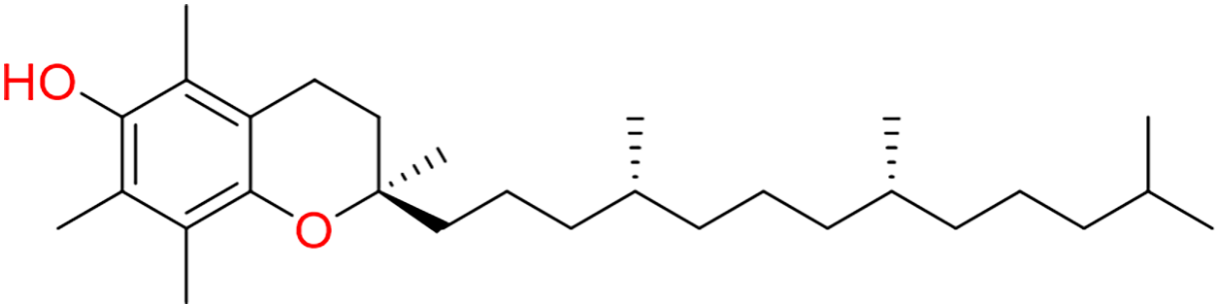	Peel Pulp	Antioxidant, anti-inflammatory, antidiabetic, anticancer	La Torre et al. (2023), Mubarak et al. (2023), de Oliveira et al. (2023)
3,4-Dihydroxybenzoic acid (protocatechuic acid)	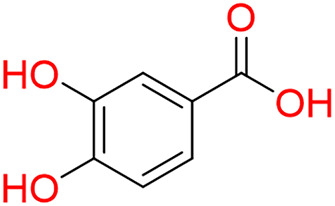	Peel	Antioxidant, antibacterial, antidiabetic, anticancer, anti-inflammatory, anti-cardiovascular diseases	Cui et al. (2023), Cadena-Iñiguez et al. (2024), Vincent, Stanely & Ponnian (2024)
Ascorbic acid	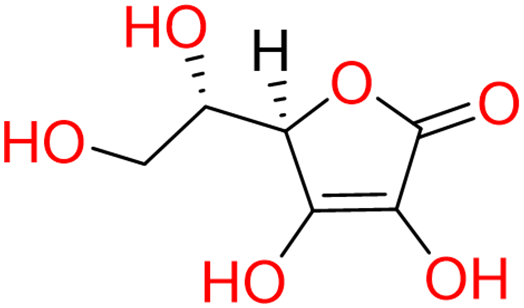	Peel Pulp Seed	Antioxidant, anti-inflammatory, antidiabetic, cardioprotective, anticancer	Gupta et al. (2022), Ali et al. (2024), Mahdavi et al. (2024), Yi et al. (2024), Mustafa et al. (2025b)
Linalool	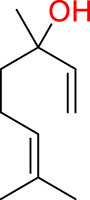	Pulp	Antioxidant, anticancer, neuroprotective, anti-inflammatory	An et al. (2023), Hosseini et al. (2023), Goswami et al. (2025)
Catechin	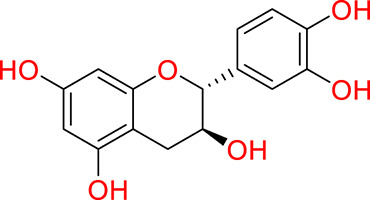	Pulp Peel Seed	Antioxidant, anti-inflammatory, antimicrobial, antidiabetic	Alañón et al. (2021b), Zhao et al. (2022), Chan et al. (2023), Bulut et al. (2025), Duarte et al. (2025), Patel et al. (2025)
Mangiferin	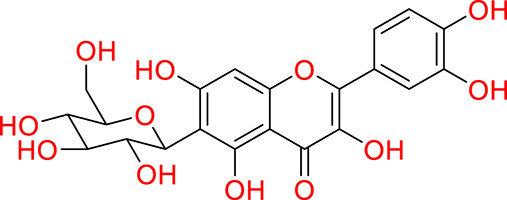	Pulp Peel Seed	Antioxidant, anti-inflammatory, antidiabetic, cardioprotective, anticancer, antimicrobial, immunomodulatory, neuroprotective, hepatoprotective, nephroprotective	Samadarsi et al. (2022), Mistry et al. (2023), Cheng et al. (2025), Duarte et al. (2025), Mustafa et al. (2025a)
Lutein	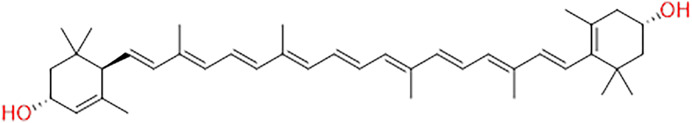	Pulp Peel	Antioxidant, anti-inflammatory, photoprotective, neuroprotective, cardioprotective, anticancer	Miyake et al. (2014), Liang et al. (2020), Chen et al. (2021), Różanowska et al. (2021), Chew et al. (2022), Shi et al. (2024)

It is important to note that the majority of reported biological effects of mango-derived bioactive compounds are based on *in vitro* assays or animal models. Although these studies provide valuable mechanistic insights, their direct translation to human health outcomes remains limited. Clinical evidence supporting these effects is still scarce, and further well-designed human intervention studies are required to confirm their efficacy and bioavailability under physiological conditions.

### Antioxidant activity

Antioxidant capacity is one of the most consistently reported functional features of mango by-products, with mango peel standing out due to its combined nutritional and phytochemical value. Beyond providing dietary fibre and macronutrients, the peel is particularly rich in phenolic compounds, flavonoids and carotenoids. The antioxidant behaviour of phenolics is closely linked to their hydroxylated aromatic structures, which favour hydrogen donation and radical quenching, yielding more stable reaction products. Compounds frequently described in mango peel include gallic acid, catechin derivatives (*e.g*., epicatechin gallate and epigallocatechin gallate), flavonols such as kaempferol and quercetin derivatives, rutin, mangiferin and procyanidins. Carotenoids further reinforce the antioxidant profile of the peel (Gómez-Caravaca et al., 2016; Gupta et al., 2022; Tacias-Pascacio et al., 2022).

Characterising antioxidant constituents is essential to support the valorisation of mango by-products. In mango peel, TPC has been shown to correlate strongly with ABTS radical-scavenging activity (r = 0.961) when comparing cultivars such as Tommy Atkins, Haden and Kent. Across these samples, gallic acid and rutin were consistently identified, whereas mangiferin peaked in Tommy Atkins (314 mg/100 g). Evidence from microwave-assisted extraction (MAE) further reinforces the high antioxidant potential of peel extracts, with a DPPH value of 6.47 μg/mL reported for MAE-derived samples (Marcillo-Parra et al., 2021; Sánchez-Camargo et al., 2021).

Using dried mango peel, extracts have also been described as phenolic-rich (TPC: 723.2 ± 0.93 mg/kg dry peel) and highly active in multiple assays, including DPPH (92 ± 4.2% scavenging), ABTS (79 ± 2.5% inhibition) and FRAP (4.7 ± 0.5 μM TE/kg dry peel). *In vitro* assays further indicated an IC50 of 15 mg/mL, and the phenolic fraction showed enhanced cytotoxicity against A549 lung cancer cells (Bai et al., 2018).

The bioaccessibility-related behaviour of phenolics from Ataulfo mango peel was investigated by Pacheco-Ordaz et al. (2018), who examined intestinal permeability alongside cellular antioxidant responses across several extract types. Their results revealed that acid and alkaline hydrolysis could release bound phenolic compounds such as gallic acid, ethyl gallate, mangiferin, and quercetin. Both the acid and alkaline hydrolysis fractions exhibited significant cellular antioxidant activity, measured by dichloro-dihydro-fluorescein diacetate fluorescence assay reaching 60.5% and 51.5% respectively at 125 µg/mL underscoring the high antioxidant potential of these compounds. Moreover, the study measured the apparent permeability coefficient (Papp) of gallic acid from the alkaline fraction across intestinal cell monolayers (Caco-2/HT-29), obtaining a value of 2.61 × 10^−6^ cm/s, which is comparable to that of pure gallic acid (2.48 × 10^−6^ cm/s). These findings indicate not only that mango peel-derived phenolics possess potent antioxidant activity, but also that they exhibit favorable bioaccessibility, highlighting their potential for use in nutraceutical formulations aimed at promoting health.

Additionally, in another study using mango peel powder to make muffins, the extracts of the mango peel powder demonstrated anti-inflammatory activity in a Caco-2 cell assay. At concentrations of 10, 50, and 100 μg/mL, the extract significantly inhibited IL-8 secretion (6.35%, 27.47%, and 61.56%, respectively) and reduced ROS production (28.02% and 61.91% at 50 and 100 μg/mL) as well as TNF-α levels (29.31% and 65.93% at 50 and 100 μg/mL) while maintaining cell viability (Plaitho et al., 2024).

Building on these promising *in vitro* results, researchers extended their investigations to animal models. In one study focused on the antidiabetic characteristics of mango peel, streptozotocin-induced diabetic rats received a diet supplemented with mango peel. This intervention effectively mitigated the development of diabetic cataracts. The protective effect was primarily attributed to an enhancement in antioxidant defense mechanisms exposing their antioxidant properties, coupled with the inhibition of polyol pathway enzymes and the suppression of advanced glycation end products formation in the lenses of diabetic rats (Gondi et al., 2017).

Further extending the *in vivo* research in animals, another study examined the impact of mangiferin supplementation in Wistar rats. Here, mangiferin produced a dose-dependent activation of antioxidant enzymes by upregulating the Nrf2 pathway which is a critical mechanism in the cellular defense against oxidative stress. As a result, the supplementation maintained a healthy balance between beneficial antioxidants and potentially harmful oxidants, thereby effectively counteracting oxidative stress (Li et al., 2020).

Moreover, another study conducted on Wistar rats investigated the anti-inflammatory and antioxidant effects of hydroalcoholic extracts from mango peel and pulp in a rat model of naproxen-induced gastric injury. The extract not only prevented oxidative damage in gastric tissue, as evidenced by significantly reduced myeloperoxidase activity and malondialdehyde content, but also maintained glutathione levels near those of the control. Notably, treatment hydroalcoholic extracts of mango peel (30 mg/kg) and mango pulp (10 mg/kg) prevented naproxen-induced glutathione decline and restored its content to normal levels, effectively neutralizing oxidizing agents and reducing the severity of gastric lesions (Ferreira Gomes et al., 2022).

Overall, these studies underscore the dual role of mango peel-derived phenolics as potent antioxidants agents, highlighting their substantial potential for incorporation into nutraceutical and functional food applications. This approach presents an eco-friendly opportunity to valorize agro-industrial waste (Tacias-Pascacio et al., 2022).

### Antidiabetic activity

Diabetes is a major public health concern worldwide. It is the seventh leading cause of death globally. This progressive disease affects 34.2 million individuals in the United States and 463 million worldwide (Saeedi et al., 2019; Zarasvand et al., 2023). Such prevalence necessitates a comprehensive and accurate approach to treatment. Current methods of managing diabetes are expensive and may cause harmful side effects. As a result, ongoing research is focused on the discovery of plant-based food products and their bioactive compounds, which are being investigated as attractive alternatives due to their cost-effectiveness, reduced side effects, and lower toxicity. These products could be beneficial in preventing type 2 diabetes mellitus (*e.g*., in individuals with prediabetes) and/or as a complementary therapy (Gupta et al., 2022; Zarasvand et al., 2023). Furthermore, mango contains many polyphenols and nonpolyphenolic bioactive compounds. Moreover, numerous studies show that the dietary fiber found in mango by-products can help to lower postprandial glycemia by delaying the rate of dietary glucose absorption (Wall-Medrano et al., 2020). The availability, cost, and nutraceutical content of mango suggest that it could be a promising antidiabetic agent.

Lasano et al. (2019) evaluated the effects of different mango peel and mango seed kernel extracts on the inhibition of enzymes related to diabetes activity; α-amylase and α-glucosidase inhibition test. Their results revealed that the mango seed kernel extract, at concentrations ranging from 2.67 to 9.83 mg/mL, exhibited a significantly stronger inhibitory effect on α-amylase than the extract directly derived from the peel. Furthermore, among all the extracts tested, the ethanol extract from the peel demonstrated the highest level of α-amylase inhibition. According to Lasano et al. (2019) the most prevalent compounds responsible for the antidiabetic action of mango peel and mango seed kernel are phenolic acid, ellagic acid, and flavonoids. Rodríguez-González et al. (2017) examined the mechanisms underlying the antidiabetic effects of mango by-products, specifically peels and leftover pulp in 3T3-L1 adipocyte cells. Their study indicates that the enhanced glucose uptake observed in insulin-dependent tissues may be attributed to the high levels of polyphenols and carotenoids present in these by-products. When compared to a negative control, treatment with mango by-product extracts at 150 μg/mL markedly increased the expression of Irs1, Pi3k, and Glut4. In fact, both the polyphenol- and carotenoid-rich extracts raised Irs1 expression by approximately 1.7–1.9-fold relative to controls. Furthermore, the polyphenol extract exhibited the strongest effect on Pi3k, promoting a threefold increase, while the carotenoid extracts significantly boosted Glut4 expression by 2.6-fold. These changes in gene expression suggest that the bioactive compounds not only mimic insulin effect but also enhance glucose uptake in adipose tissue a mechanism that may explain their hypoglycemic effects observed *in vivo* (Rodríguez-González et al., 2017).

Animal studies offer further support for the antidiabetic potential of mango derivatives. mango peel has been assessed in a small number of studies using STZ-induced diabetic rats (Parmar & Kar, 2008; Gondi et al., 2017; Preciado-Saldaña et al., 2022). Preciado-Saldaña et al. (2022) investigated the hypoglycemic properties of Ataulfo mango peel hydroethanolic (20:80) extracts using both *in vitro* enzyme inhibition assays (α-amylase and α-glucosidase inhibition assay) and an *in vivo* prediabetic rat model. Their results showed that the extracts not only normalized blood glucose and lipid levels compared to healthy and unsupplemented prediabetic rats but also exhibited enzyme inhibitory activity with an IC_50_ of approximately 0.085 mg/mL. These findings suggest that the consumption of mango peel extracts during the prediabetic phase may help prevent the onset of type 2 diabetes (Preciado-Saldaña et al., 2022).

One notable study investigated the effects of mango juice enriched with mango peel and residual pulp on streptozotocin-induced diabetic Wistar rats, aiming to elucidate the mechanisms behind its antidiabetic properties (Rodríguez-González et al., 2017). The enriched juice significantly reduced serum glucose levels (*p* < 0.05) in these diabetic rats, although this effect was not associated with decreased intestinal glucose absorption or protection of the Langerhans islets. In parallel, *in vitro* experiments with 3T3-L1 adipocyte cells demonstrated that mango juice exerts insulin-mimetic actions by upregulating the expression of Glut4, Irs1, and Pi3k. Additionally, mango by-products significantly lowered serum triacylglycerides (*p* < 0.05) in diabetic rats, an outcome linked to reduced intestinal lipid absorption, and alleviated diabetic nephropathy through its renal antioxidant activity. These antidiabetic effects appear to stem from the high content of soluble fiber, as well as several bioactive compounds. Consequently, these findings suggest that mango by-products could serve as a valuable functional supplement for diabetes management.

In human clinical trials, there is a research investigating the potential of mango using mango fruit powder to improve glucose metabolism and endothelial function. Seventy-five men and women in the early stage of impaired glucose metabolism received daily doses ranging from 100 to 300 mg of mango powder. The study found significant improvements in HbA1c levels and blood flow, likely due to enhanced microcirculation, as well as improved postprandial endothelial function, which may be attributed to the increased of the antioxidant activity and the enhanced glues metabolism (Buchwald-Werner et al., 2017). Notably, there is no available data from any study using mango peel in human subjects.

### Anticancer activity

Cancer remains one of the foremost causes of global mortality, making the discovery of effective treatments critically important for public health (Mirza et al., 2021; Gupta et al., 2022). A range of phytochemical compounds found in various parts of the mango such as gallic acid, mangiferin, quercetin, isoquercetin, pentagalloyl glucose, and gallotannins has been shown to possess potent free radical scavenging capabilities as well as strong cytotoxic effects against several cancer cell lines, including those from blood, lung, breast, colon, and prostate cancers (Zhao et al., 2014; Rajeshkumar et al., 2018; Lauricella et al., 2019; Kumkoon et al., 2024). In addition to these phenolic compounds, carotenoids, particularly β-carotene, have long been associated with cancer prevention. As early as 1981, Peto et al. (1981) reported an inverse correlation between human cancer risk and both blood retinol and dietary β-carotene intake (Peto et al., 1981), prompting numerous subsequent intervention studies to explore this relationship. Recent reviews further reinforce the anticancer potential of carotenoids, highlighting their ability to modulate oxidative stress and signalling pathways involved in tumor progression (Ojobor et al., 2025).

In particular, Kumkoon et al. (2024) investigated the antioxidant effect and anti-breast cancer properties of mango peel extracts derived from Bao cultivar. The study demonstrated that the extracts exerted a significant inhibitory effect on MDA-MB-231 breast cancer cells, yielding an IC_50_ of 39.08 ± 5.22 µg/mL. Furthermore, the researchers successfully developed nanoencapsulated formulations of the mango peel extract by employing chitosan-coated poly lactic-co-glycolic acid. These nanoformulations exhibited a spherical morphology with an average diameter of 251.90 ± 19.61 nm and an encapsulation efficiency of 30.48%. Notably, the nanoencapsulated extract showed enhanced anticancer activity compared to the free extract, as evidenced by its greater impact on reducing cell viability and inhibiting the migration and invasion of the breast cancer cells. Collectively, these findings suggest that both mango peel extracts and their nanoencapsulated counterparts hold promise as viable alternatives to conventional chemical agents for breast cancer treatment.

In another investigation, researchers prepared mango peel extracts using solvents of varying polarity to isolate distinct active phytochemicals. This study not only profiled the antioxidant capabilities of the extracts but also explored their antibacterial activity against foodborne pathogens, their anti-biofilm potential, and their *in vitro* anticancer effects against colon cancer. Among the various extracts, the ethyl acetate fraction emerged as the most phytochemically enriched. At a concentration of 500 µg/mL, this extract exhibited complete bactericidal action against foodborne pathogens as determined by minimum inhibitory concentration and minimum bactericidal concentration values while lower concentrations (125–250 µg/mL) did not display antibacterial activity. Moreover, the ethyl acetate mango peel extract showed anticancer activity against the human colon epithelial cell line ATB-37 (Caco-2) with an IC_50_ of 430.36 µg/mL. Complementary molecular docking analyses further revealed that the major phytochemicals achieved top-ranked binding conformations with key targets such as cyclooxygenase-2 (COX2, P00406) and nuclear factor kappa B (NFKB, Q63369), thus providing insight into the molecular basis of their bioactivity (El-Sayed et al., 2025).

Additional research by Lauricella et al. (2019) focused on the anticancer properties of an ethanolic mango peel extract against human colorectal cancer cell lines (Caco-2, HCT116, and HT-29). The extract not only reduced cell viability and induced noticeable morphological changes but also inhibited tumor colony formation. These effects were attributed to the induction of DNA fragmentation and apoptotic cell death, processes driven by an increase in reactive oxygen species (ROS), activation of the JNK and ERK1/2 pathways, and an upregulation of antioxidant defenses, including manganese superoxide dismutase (MnSOD) and nuclear factor erythroid 2-related factor 2 (Nrf2).

Mangiferin, another prominent mango-derived compound, has also been investigated for its anticancer effects. For instance, Deng, Tian & Liang (2018) reported that mangiferin inhibited the growth and metastasis of breast cancer cells including MCF-7 and MDA-MB-231 in a concentration-dependent manner. This inhibition was linked to the suppression of multiple mitogenic and metastatic signalling pathways. In particular, mangiferin significantly impaired the migration and invasion of MDA-MB-231 cells by disrupting the Rac1/WAVE2 signalling axis, as reflected by decreased levels of actin-related proteins (Arp2, Arp3), WAVE2, Rac1/Cdc42, and phosphorylated Rac1 (Deng, Tian & Liang, 2018; Mei, Ma & Chen, 2021).

Complementing the *in vitro* results, *in vivo* studies further validate the anticancer potential of mango in animal models. In recent studies, Shaban et al. (2023) investigated the therapeutic effects of mango seed kernel extracts, mango peel extracts, and their combination over 4 weeks in rats with mammary tumors induced by 7,12-dimethylbenz[a]anthracene (DMBA). The treatments effectively and safely reduced tumor progression and associated tissue damage.

The authors reported that these phenolic- and flavonoid-rich extracts effectively suppressed tumor development and mitigated tissue damage without causing observable adverse effects. Mechanistically, their protective effects were attributed, at least in part, to the attenuation of oxidative stress through the enhancement of endogenous antioxidant defenses, including superoxide dismutase, glutathione S-transferase, glutathione reductase, total glutathione peroxidase, and reduced glutathione. In addition, the extracts exerted anti-estrogenic and antiproliferative effects by downregulating ER-α expression and reducing E2 levels, while concurrently promoting apoptosis in malignant cells, as evidenced by increased DNA fragmentation and caspase-3 activity. Overall, the findings support the notion that mango extracts, rich in phenolics and flavonoids, offer antioxidant, antiproliferative, proapoptotic, and anti-estrogenic benefits, underscoring their promise as adjuncts in pharmacological strategies against DMBA-induced mammary tumors.

Despite the promising results observed in both *in vitro* and *in vivo* studies, clinical evidence regarding the anticancer effects of mango in humans remains scarce but there is evidence from bioactive compounds isolated from mango. Recent research has highlighted the potential of mango derived extracts such as those from the peel and seed kernel or the potential of specific isolated compounds like mangiferin or quercetin to exert antiproliferative, proapoptotic, and antioxidant effects in various cancer models. These effects are mediated through mechanisms including the induction of apoptosis, inhibition of cell migration and invasion, and modulation of key signalling pathways such as JNK, ERK1/2, and Rac1/WAVE2. *In vivo* studies have further supported these findings by demonstrating reduced tumor progression and oxidative stress in animal models. However, no clinical trials have been conducted in recent years to validate these findings in human populations, limiting the translational potential of these results. Therefore, well-designed clinical studies are urgently needed to assess the efficacy and safety of mango-based interventions in oncology and to determine their viability as functional foods or therapeutic agents.

### Anti-inflammatory activity

Inflammatory bowel disease, characterized by chronic intestinal inflammation and microbial dysbiosis, is a major risk factor for the development of colorectal cancer (Kim et al., 2020). Inflammation plays a central role in cancer progression, contributing to the proliferation, invasion, and metastasis of tumor cells. Chronic inflammation can promote carcinogenesis in various tissues through multiple molecular pathways (Greten & Grivennikov, 2019).

At the molecular level, inflammation involves the activation of nuclear factor kappa (NF-κB) signalling pathways, upregulation of pro-inflammatory enzymes such as cyclooxygenase-2 (COX-2) and inducible nitric oxide synthase (iNOS), and increased expression of cytokines including tumor necrosis factor-alpha (TNF-α), interleukins IL-6, IL-8, IL-1β, and chemokines such as CCL2 and CXCL8 (Li et al., 2016; Mei, Ma & Chen, 2021).

Bioactive compounds derived from mango by-products, such as mangiferin, phenolic compounds and carotenoids such as lutein and β-carotene found in the peel, seed, and pulp, have demonstrated anti-inflammatory activity by inhibiting key pro-inflammatory enzymes such as COX-2 and lipoxygenase. Additionally, these compounds modulate the NF-κB signalling pathway, thereby reducing the expression of inflammatory mediators (Naraki et al., 2021; Duarte et al., 2025). Mangiferin, in particular, has been shown to suppress the production of cytokines such as IL-6, IL-10, and TNF-α, contributing to the attenuation of inflammatory responses (Mei, Ma & Chen, 2021; Gupta et al., 2022; Duarte et al., 2025).

*In vitro* studies further support these findings. Arbizu-Berrocal et al. (2019) demonstrated that mango polyphenols modulate the expression of microRNAs associated with the PI3K/AKT/mTOR signalling pathway, which is closely linked to inflammation and cell survival. In non-cancerous breast epithelial cells (MCF-10A), treatment with mango polyphenols significantly reduced the expression of pro-inflammatory markers, suggesting a protective effect against inflammation-induced cellular stress.

Extending the findings from *in vitro* studies, *in vivo* research has confirmed the anti-inflammatory potential of mango. Ferreira Gomes et al. (2022) investigated the antioxidant and anti-inflammatory effects of hydroalcoholic extracts from mango peel and pulp (Tommy Atkins) in a naproxen-induced gastric injury rat model. The treatment significantly reduced myeloperoxidase activity and malondialdehyde levels, both of which are key biomarkers of inflammation and lipid peroxidation. Additionally, the extracts preserved glutathione levels, indicating enhanced antioxidant defence mechanism.

In a related study, Paradee et al. (2023) evaluated the biological activity of mango pulp extracts in murine models. The extract demonstrated strong antioxidant capacity and effectively reduced inflammation-related biomarkers. Moreover, the extracts exhibited protective effects against gastric ulcers induced by stress, hydrochloric acid/ethanol, and indomethacin. These results suggest that mango bioactives show a multifaceted protective role, combining antioxidant, anti-inflammatory, analgesic, gastroprotective, and hepatoprotective properties.

In addition to *in vitro* and animal studies, emerging clinical evidence supports the anti-inflammatory potential of mango polyphenols in humans. In a pilot study involving patients with inflammatory bowel disease, daily supplementation with mango polyphenols resulted in a significant reduction in plasma levels of pro-inflammatory cytokines, including interleukin-8, growth-regulated oncogene, and granulocyte-macrophage colony-stimulating factor. Furthermore, the intervention was associated with an increase in beneficial gut microbiota, particularly Lactobacillus species, suggesting a dual anti-inflammatory and microbiota-modulating effect. These findings highlight the potential of mango-derived polyphenols as a dietary strategy to support gut health and manage chronic intestinal inflammation in inflammatory bowel disease patients (Kim et al., 2020). These results underscore the potential of mango-derived polyphenols not only in cancer prevention but also in the regulation of inflammatory processes at the molecular level.

### Sustainable applications of mango by-products in the juice industry

The global mango processing industry generates substantial quantities of by-products, particularly mango peels, which account for approximately 15–25% of the fruit’s weight and with mango seed kernels it will constitute 40% of the fruits weight (Serna-Cock, García-Gonzales & Torres-León, 2016; Rojas et al., 2020). As stated in the introduction of the review, mango peel, which is traditionally discarded as waste, has been increasingly recognized for its richness in bioactive compounds such as polyphenols, carotenoids or dietary fiber (Serna-Cock, García-Gonzales & Torres-León, 2016; Castro-Vargas et al., 2019; Villacís-Chiriboga et al., 2021; Tacias-Pascacio et al., 2022). However, the exclusion of these valuable components from juice production can significantly diminish the nutritional and functional quality of the final product.

As consumer demand for functional beverages with minimally processed and environmentally sustainable food systems continues to grow, the trend towards clean labels has accelerated research and the adoption of non-thermal technologies that can deliver microbial stability while preserving nutrients and sensory quality (Zia et al., 2024). Moreover, the valorization of mango by-products, particularly the peel, into juice manufacturing presents a promising opportunity to enhance nutritional value, reduce environmental impact, and improve circular economy practices within the food industry (Zhang et al., 2019; Tacias-Pascacio et al., 2022; Kaur, Panesar & Thakur, 2025). This approach aligns with broader sustainability goals and supports innovation in the development of health-promoting beverages.

### Functional potential in juice formulations

Mango pulp, being the main consumable part of the fruit, is already widely used in juice production due to its appealing flavour and high nutrient content (Lebaka et al., 2021). Given that mango contains two provitamin A carotenoids (β-carotene and β-cryptoxanthin) it can also contribute to vitamin A intake. Nevertheless, recent studies have shown that mango peel contains significantly higher levels of phenolic compounds and carotenoids compared to the pulp (Martínez-Ramos et al., 2020; Tacias-Pascacio et al., 2022; Kučuk et al., 2024; Rodríguez-Rodríguez et al., 2025). Incorporating mango peel extracts or powders into juice formulations can significantly boost the antioxidant capacity of the final product, contributing to health benefits such as anti-inflammatory and antidiabetic effects (Gondi et al., 2017; Rodríguez-González et al., 2017; Plaitho et al., 2024).

For instance, mango peel extracts have shown strong radical scavenging activity and antimicrobial properties (Bai et al., 2018; El-Sayed et al., 2025), making them promising ingredients for both enhancing nutritional value and extending the shelf life of juice products. Moreover, the addition of mango peel-derived fiber can improve the functional profile of juices by increasing dietary fiber content, which is often lacking in fruit beverages (Ajila & Prasada Rao, 2013; Garcia-Amezquita et al., 2018). Recent research has also focused on the valorization of mango by-products during juice processing. For example, Cruz-Cansino et al. (2023) optimized ultrasound-assisted extraction conditions using response on mango (Ataulfo) by-products comprising peels, pulp residues and seeds to maximize the recovery of antioxidant compounds such as TPC, ascorbic acid (AA) and antioxidant activity (ABTS and DPPH assays). The study identified optimal parameters of 91% of amplitude and 7 min, which yielded the highest extraction efficiency for TPC, AA, and antioxidant activity, demonstrating that ultrasound is a promising method to enhance the extraction of antioxidants from mango industrial waste. These ultrasound-assisted extracts, rich in bioactive compounds, could also be directly incorporated into mango juice formulations to enhance their functional properties and nutritional value. In this context, although mango peel is increasingly valorized as a source of bioactive compounds, it is important to consider safety aspects. Mango peel and sap contain urushiol-like alkylresorcinols, compounds structurally related to those found in poison ivy, which may cause contact dermatitis in sensitive individuals. However, these compounds are primarily localized in the peel, and their presence in the edible pulp and conventional mango juice is considered negligible (Yoo & Carius, 2019; Alipour Tehrany & Coulombe, 2021). Most reported adverse reactions are associated with direct skin contact rather than oral consumption. Additionally, as with other tropical fruits, pesticide residues may be detected if good agricultural and post-harvest practices are not followed. Therefore, compliance with maximum residue limits and appropriate monitoring is essential, particularly when by-products such as peel are incorporated into functional formulations.

In addition, mango juice has also been explored as a carrier for probiotic microorganisms. Mousanejadi et al. (2023) investigated the application of *Lactobacillus casei* UK 318, a strain isolated from Iranian yogurt, in mango juice. The strain demonstrated a strong probiotic potential including acid and bile tolerance, antioxidant activity, cholesterol removal, and high antimicrobial effects on pathogenic bacteria. When incorporated into mango juice at concentrations of 0–2%, it maintained high viability during 21 days of storage, particularly at 4 °C, and achieved the highest sensory acceptance and lowest color change and the highest viable cell count at 2% concentration. These findings highlight mango juice as a promising matrix for the development of probiotic beverages with enhanced functional and sensory properties. Moreover, other studies have explored the impact of different bacterial fermentations on the volatile and nutrient composition of mango juice, further supporting its potential in functional beverage innovation (Liu et al., 2023b, 2025; Zhong et al., 2023; Guo et al., 2025).

### Impact of processing technologies on mango juice quality

Mango juice is highly valued for its richness in vitamin C, carotenoids, and polyphenols, compounds associated with health benefits but highly sensitive to heat. Processing aims to ensure microbial safety and storage stability while preserving nutritional and sensory quality. Traditionally, these objectives have been achieved through thermal pasteurization, which is effective against microorganisms and enzymes but also degrades heat-sensitive bioactive compounds such as vitamin C, carotenoids, and polyphenols, reduces their bioaccessibility, and alters the volatile profile and antioxidant activity, thereby compromising the nutritional and functional integrity of the final product (Zhang et al., 2019; Wongkaew et al., 2021b; Tacias-Pascacio et al., 2022). Furthermore, Silva et al. (2022) demonstrated that thermal processing in mango pulp not only reduces bioactive compounds but also alters the volatile profile and the antioxidant activity, negatively impacting its sensory and functional properties despite ensuring microbial safety.

Conversely, literature reviews focusing on fruit-based products consistently emphasize non-thermal technologies such as high-pressure processing (HPP), high-pressure homogenization (HPH), pulsed electric fields (PEF), and ultraviolet (UV) treatment as effective alternatives for preserving quality and safety (Gupta et al., 2023; Zia et al., 2024). Among these, HPP stands out as it is already commercially implemented at an industrial scale for fruit juices and beverages, offering a validated solution for microbial safety without compromising nutritional or sensory quality (Barbosa-Cánovas et al., 2025). HPP applies pressures of 200–600 MPa at ambient or chilled temperatures, minimizing thermal degradation (Fernández-Ponce et al., 2015; Mahadevan & Karwe, 2016; Al-Juhaimi et al., 2018). This technology is particularly attractive because it extends shelf life, preserves fresh-like characteristics, and meets clean-label demands, which are key drivers in the functional beverage market (Barbosa-Cánovas et al., 2025). Studies have shown that HPP-treated mango juice retains significantly higher levels of vitamin C, total phenolics, and antioxidant activity compared to thermally processed juice (Zhang et al., 2019). Bi et al. (2020) further demonstrated that HPP at 500 MPa for 8 min effectively inactivated microorganisms in mango smoothies while better preserving carotene content, color, and viscosity during refrigerated storage, compared to both thermal treatment and others HPP conditions.

Beyond microbial safety and quality retention, recent research has focused on the nutritional functionality of mango juice, particularly the digestive fate of bioactive compounds. Hu et al. (2023a) explored the effect of HPP and HPH on the bioaccessibility and gastric retention rate of carotenoids in mango juice. Compared to the control, HPP treatments specifically at 400 MPa on samples pre-treated 100 MPa HPH significantly increased the bioaccessibility by 71.37% and the gastric retention rate by 24.24%. They highlight that the pectin-carotenoid interactions, as well as the solubility and dispersibility of carotenoids, may be important factor influencing the digestive fate of these compounds in juice. In a related study by the same authors, Hu et al. (2023b) focused on the effects of HPP and HPH on water-soluble pectin and its structural changes in mango juice. They found out that HPH at 50 and 100 MPa had a stronger effect than HPP on water-soluble pectin, increasing its molecular weight and the concentrations of pectin and galacturonic acid in the mango beverage, while reducing its degree of methylesterification. These structural changes in water-soluble pectin were positively correlated with carotenoid bioaccessibility, highlighting the role of pectin-carotenoid interactions in digestion. Such findings underscore the potential of combining HPP and HPH not only for ensuring safety and extending shelf-life, but also for enhancing the functional value of mango-based beverages.

Moreover, beyond nutritional and functional aspects, sensory quality is a critical determinant of consumer acceptance. HPP has been reported to better preserve the sensory attributes of mango juice, such as color and aroma, which are often compromised during heat treatment (Zhang et al., 2019). This advantage is possibly linked to the minimal degradation of volatile compounds under pressure, as compared to heat-induced degradation, resulting in a more authentic sensory profile during storage (Zia et al., 2024). Such preservation is particularly relevant for juice formulations enriched with mango peel extracts or other functional ingredients, where maintaining both the bioactive and aromatic profile is essential to deliver health benefits along with a desirable sensory experience (Tacias-Pascacio et al., 2022). Consequently, HPP aligns with the growing demand for premium, clean-label beverages that combine functionality with sensory appeal.

In addition to HPP, other non-thermal processing technologies have been explored in research and can be applied on mango juice. Pulsed electric fields (PEF) was studied as a non-thermal processing on dried mango peels at different intensities (1.5, 3.0 and 4.5 kV cm^−1^), and different dried temperatures (50 °C, 60 °C and 70 °C). The study showed that PEF resulted in an improving of the efficiency, especially applying 4.5 kV cm^−1^ and a dry at 70 °C, where they observed a reduction in the drying time of 67%, and the lowest degradation of total phenolic content (24.30%), and higher solubility (78.478%) and the higher values of antioxidant activity at DPPH assay, opening the opportunity of the incorporation of this by-product for functional ingredient (Santos et al., 2023). Ultraviolet (UV) irradiation has also been investigated as a non-thermal cold pasteurization method in order to improve quality and microbiological parameters of mango juice during cold storage. Wai et al. (2024) reported that UV irradiation at 120 J cm^−2^ achieved a 5.9 log reduction of microbial and extended the shelf life of mango juice by approximately 14 days compared to the thermally processed samples. However, in terms of quality parameters, the ultraviolet treated samples did not differ significantly in terms of the quality metrics respect thermal processed ones. Overall, these findings suggest that although several non-thermal technologies show promise, HPP remains the most robust and commercially viable option for mango juice, owing to its combined advantages in safety, quality preservation, and industrial scalability.

## Conclusions

Mango and its by-products, such as the pulp, peel, and seed kernel, are rich in health-promoting compounds, including phenolic compounds, carotenoids, vitamins, other micronutrients, and dietary fiber, which exhibit antioxidant, anti-inflammatory, antidiabetic, and anticancer activities. Their incorporation into food formulations offers a dual benefit: enhancing nutritional and functional properties while promoting sustainability through waste valorization.

Despite the promising evidence from *in vitro* and *in vivo* studies, significant challenges remain. These include optimizing green extraction methods to maximize yield and preserve bioactivity, ensuring compound stability during processing and storage, and validating health benefits through well-designed clinical trials. Addressing these gaps is essential for translating laboratory findings into safe, effective, and market-ready functional products.

The valorization of mango by-products through their incorporation into different food formulations can enhance the nutritional value of products and contribute to sustainability in the food industry. Future research opportunities include evaluating the preservation of bioactive properties, safety, sensory attributes, and the physiological benefits of mango-derived compounds.

From a technological perspective, non-thermal processing methods such as high-pressure processing offer a robust solution for ensuring microbial safety while preserving heat-sensitive compounds and sensory quality. When combined with the incorporation of mango peel, either as extracts or in lyophilized form these approaches can enhance the functional value of beverages and align with clean-label and circular economy principles. This strategy not only improves the nutritional profile of mango-based products but also reduces environmental impact, supporting eco-innovation and sustainable food systems.

Complementary to non-thermal processing technologies, green extraction techniques such as ultrasound-assisted extraction, microwave-assisted extraction, pressurized liquid extraction, supercritical fluid extraction, pulsed electric fields, and enzyme-assisted extraction represent sustainable strategies for recovering bioactive compounds from mango by-products. These methods enhance extraction yield, reduce solvent consumption, and preserve the biological activity of sensitive compounds, contributing to the comprehensive valorization of mango under circular economy principles. Their integration with non-thermal processes in the juice industry opens new opportunities for developing functional beverages with improved nutritional value and reduced environmental impact.

Future research should focus on integrating advanced extraction technologies with non-thermal processing, validating bioactive effects and bioavailability in humans, and assessing consumer acceptance. Pilot-scale and industrial studies, alongside technoeconomic and life-cycle analyses, will be crucial to ensure the sustainability, safety, and commercial feasibility of mango-derived ingredients. These efforts will pave the way for the development of next-generation functional beverages, transforming mango products into high-value, health-promoting products that meet the growing demands for quality, efficacy, and environmental sustainability.

## Additional Information and Declarations

### Data Availability

The following information was supplied regarding data availability:

This is a literature review.
